# Direct synthesis of two-dimensional Cu_3_(C_6_O_6_) kagome conjugated coordination polymer thin films *via* chemical vapor deposition

**DOI:** 10.1039/d6ra05487k

**Published:** 2026-07-22

**Authors:** Hyeonwoo Lee, Hee Cheul Choi

**Affiliations:** a Department of Chemistry, Pohang University of Science and Technology (POSTECH) Pohang 37673 Republic of Korea choihc@postech.edu

## Abstract

Chemical vapor deposition (CVD) is a promising route for device-compatible two-dimensional (2D) conjugated coordination polymer (c-CP) thin films, but control of the coordination environment during growth remains underdeveloped. Here, we report the direct CVD synthesis of 2D kagome Cu_3_(C_6_O_6_) c-CP thin films using Cu(*t*Buacac)_2_ (*t*Buacac = *tert*-butylacetoacetate) as a rationally selected metal precursor. To avoid the THQ-pillared framework previously obtained from Cu(acac)_2_, in which kagome c-CP layers are separated by axial THQ ligands, we used the bulkier Cu(*t*Buacac)_2_ precursor to modulate the coordination pathway during CVD growth. Time-dependent *ex situ* GIWAXS analysis showed no reflections associated with the THQ-pillared framework during synthesis, due to suppression of axial THQ coordination and the direct growth of face-on layered Cu_3_(C_6_O_6_). GIWAXS, HRTEM, Raman, XPS, and FT-IR analyses also support the formation of the layered Cu_3_(C_6_O_6_) c-CP structure. The Cu_3_(C_6_O_6_) thin film exhibited an optical bandgap of ∼1.29 eV and a room-temperature conductivity of ∼0.10 S cm^−1^. This work demonstrates that metal precursor geometry is important for modulating the coordination pathway during CVD growth and provides an effective strategy for accessing targeted c-CP thin films.

## Abstract 2

A bulky Cu precursor suppresses axial THQ coordination during vapor-phase growth, preventing pillar formation and enabling direct formation of layered 2D kagome Cu_3_(C_6_O_6_) c-CP thin films.

## Graphical abstract

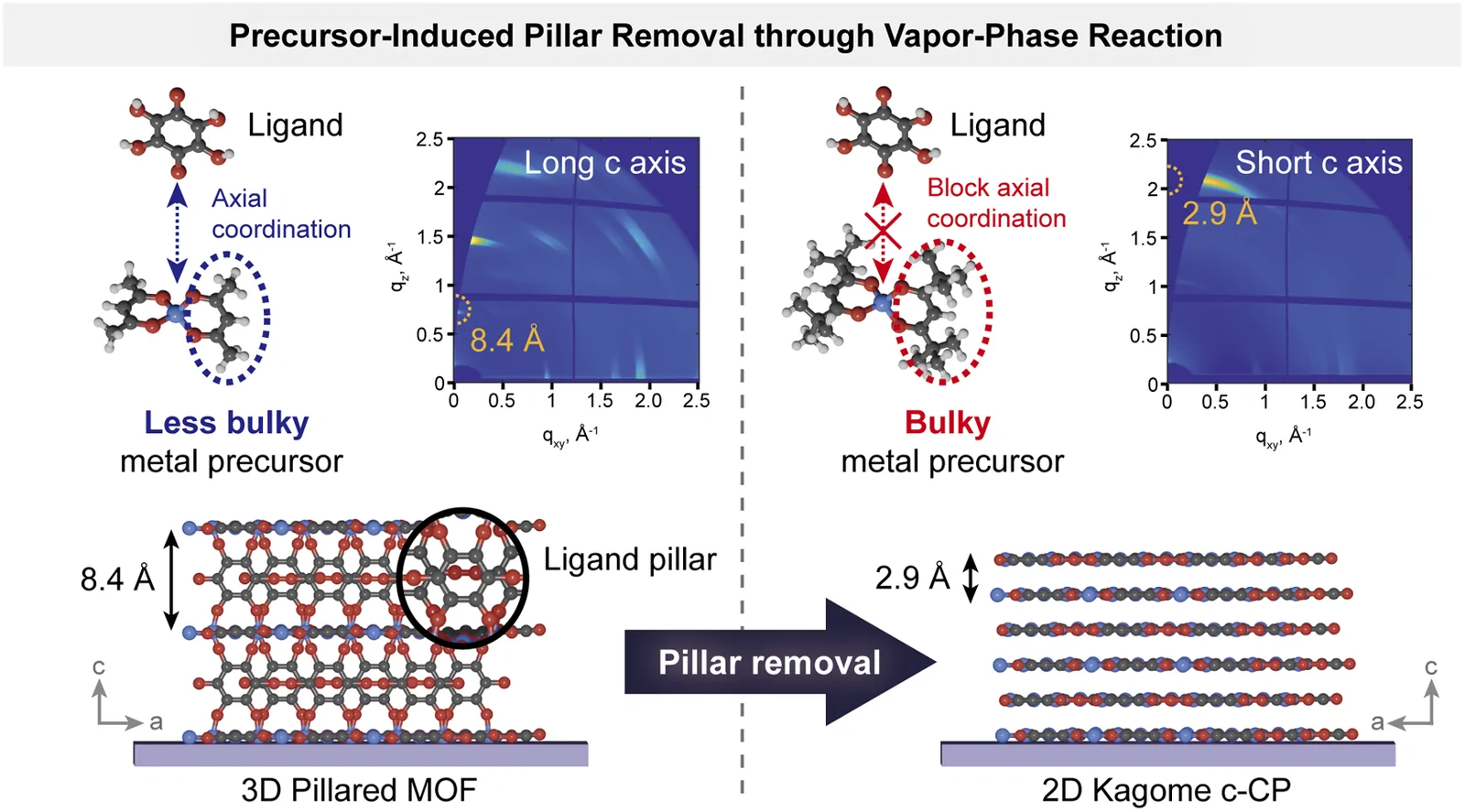

## Introduction

**Fig. 1:**
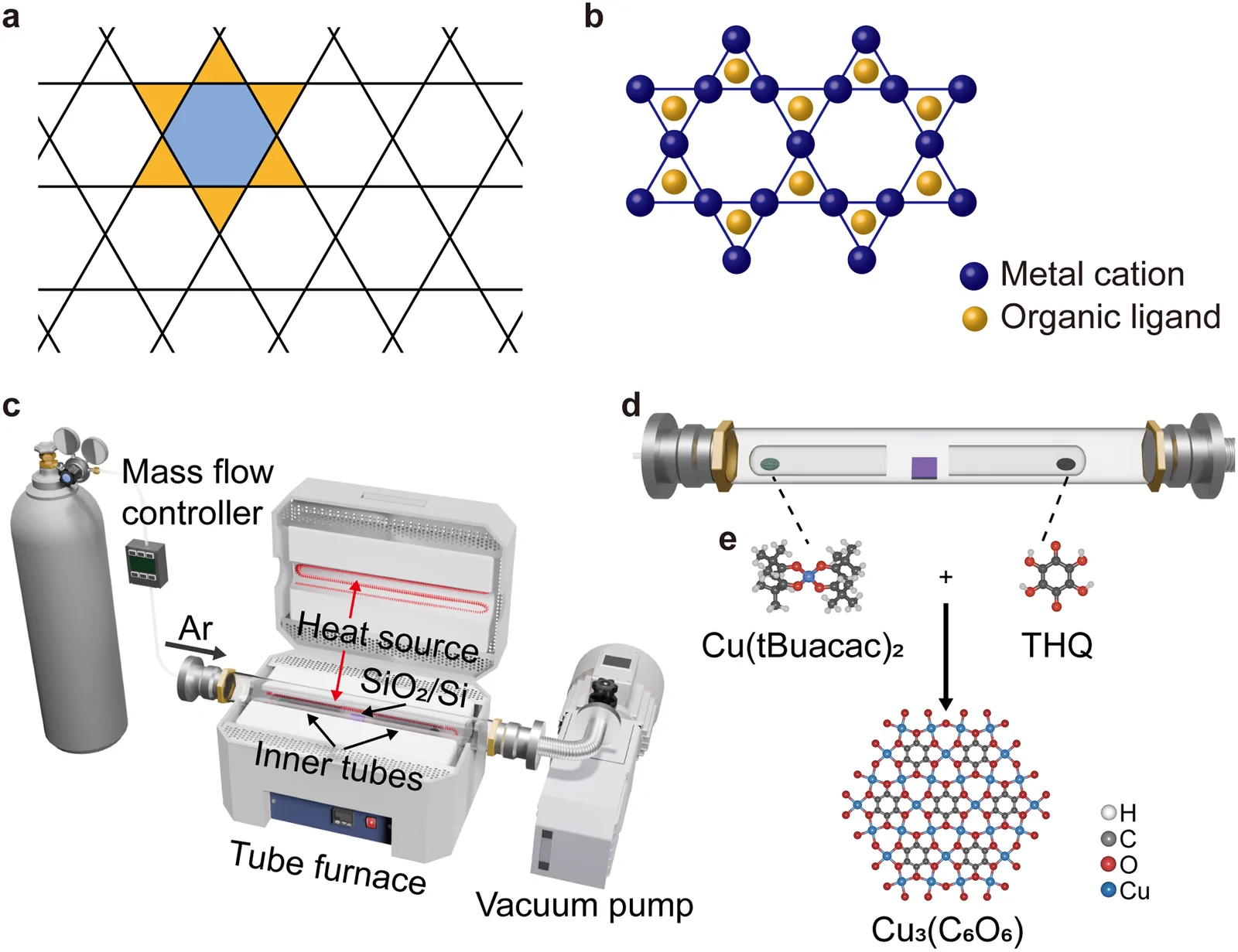
(a) Schematic illustration of a kagome lattice composed of corner-sharing triangles. (b) Schematic representation of a kagome coordination polymer formed by metal cations and organic ligands. (c) Illustration of the CVD system for Cu_3_(C_6_O_6_) film synthesis. (d) Illustration of the detailed CVD system setup for Cu_3_(C_6_O_6_) film synthesis. (e) Reaction scheme for the Cu_3_(C_6_O_6_) film synthesis.

Recently, various 2D materials with kagome structures have attracted special interest for their unique electronic properties including flat bands,^1^ Dirac cones,^2^ anomalous Hall effect^3^ and half-metallic behaviors (Fig. 1a).^4^ Two-dimensional (2D) conjugated coordination polymers (c-CPs)^5–7^ or metal–organic frameworks (MOFs)^8–11^ are potential platforms for kagome structures because they have hexagonal voids that are composed of equilateral triangles, which can be organized by the spontaneous assembly of metal cations and organic ligands (Fig. 1b).^12,13^ Their in-plane conjugation and interlayer electronic coupling enable efficient charge transport,^14–16^ making them promising material platforms for device-scale applications.^17–20^ Meanwhile, chemical vapor deposition (CVD) has emerged as a promising route to 2D c-CP thin films^21–23^ by enabling large-area deposition, thickness control, and compatibility with device fabrication. There have been many efforts to synthesize MOF or c-CP kagome-structured films by CVD,^24,25^ and the most recent and promising result has been reported by Liu *et al.* In their work, a crystalline Cu_3_(C_6_S_6_) c-CP film composed of BHT (benzenehexathiol) was synthesized with the assistance of ammonia to modulate coordination competition and crystallinity.^26^ Despite these advances, most reported single-step CVD syntheses of M_3_(C_6_X_6_)_*n*_ films (X = O or S; *n* = 1 or 2) have relied on M(acac)_*n*_ (acac = acetylacetonate) metal precursors, with precursor selection generally focused on metal identity and oxidation state. The effect of metal precursor geometry has remained relatively unexplored as a means of controlling coordination structure during vapor-phase growth.

We have recently found an interesting intermediate CP with kagome-structured layers sandwiched by THQ as pillars between the layers upon the reaction of Cu(acac)_2_ (acac = acetylacetonate) with THQ.^27^ This result suggests a possible route to fully 2D kagome Cu c-CP films if the involvement of THQ ligands as pillars can be suppressed. In view of the underexplored role of precursor geometry described above, this result further inspired us to study the effect of metal precursor geometry on the coordination pathway. This is because THQ pillars are introduced through axial coordination sites that remain accessible when Cu(acac)_2_ is used. We therefore examined whether this axial coordination can be suppressed by introducing bulkier ligands using Cu(*t*Buacac)_2_ (*t*Buacac = *tert*-butylacetoacetate) instead of Cu(acac)_2._ The bulky aliphatic group of the *t*Buacac ligand is expected to hinder axial coordination to the Cu ion.^28^

Here, we report the successful formation of a 2D kagome c-CP thin film of Cu_3_(C_6_O_6_) through CVD using Cu(*t*Buacac)_2_ metal precursor and THQ organic ligand. Structural characteristics of the resulting films were verified by grazing-incidence wide-angle X-ray scattering (GIWAXS) and high-resolution transmission electron microscopy (HRTEM) analyses. These results establish precursor design as a viable strategy for controlling structure in vapor-phase c-CP thin films and broadening the structural diversity accessible by CVD.

## Results and discussion

A home-built tube-type CVD furnace system was used to synthesize Cu_3_(C_6_O_6_) films (Fig. 1c and S1). Under an Ar atmosphere at ∼140 mtorr, Cu(*t*Buacac)_2_ and THQ were placed inside the small inner tube, which was then placed at the optimal position in the quartz tube (Fig. 1d). The furnace was heated to 140 °C and maintained for 30 minutes, and the reaction was then stopped by opening the furnace lid. The vaporized precursors met at the center where a 1 cm × 1 cm SiO_2_/Si substrate was located to form Cu_3_(C_6_O_6_) thin film (Fig. 1e and S2).

**Fig. 2:**
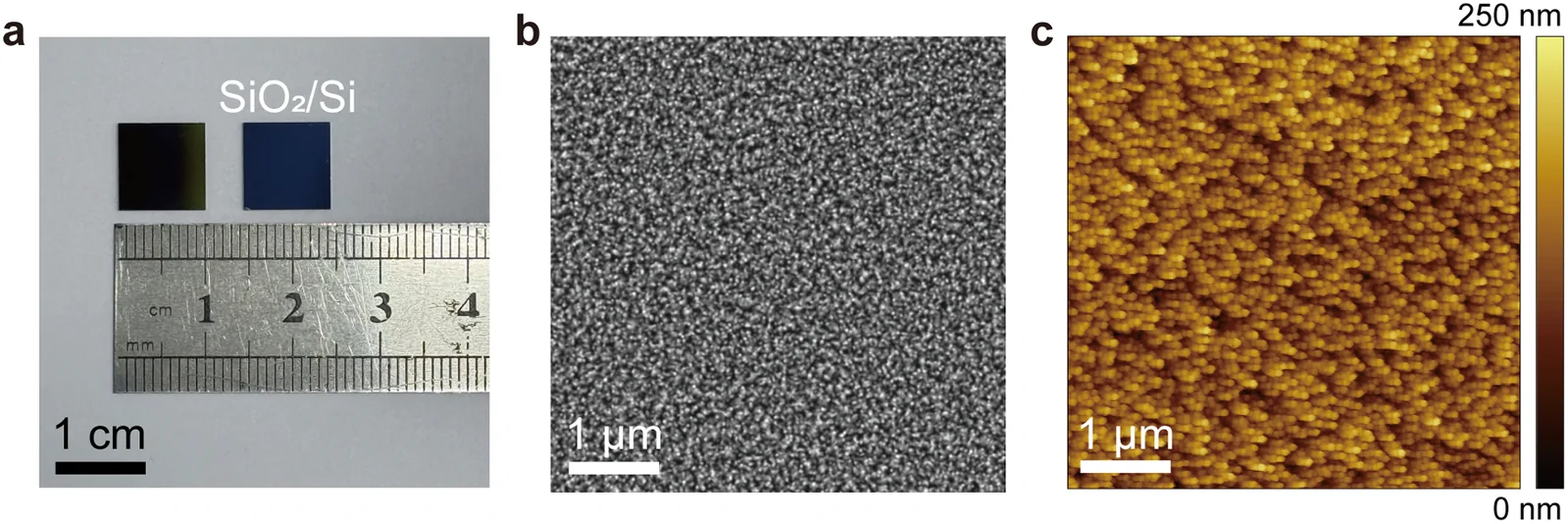
(a) Digital photograph of the Cu_3_(C_6_O_6_) film on a SiO_2_/Si substrate, shown alongside the bare SiO_2_/Si substrate. (b) SEM image of the Cu_3_(C_6_O_6_) film (c) AFM image of the Cu_3_(C_6_O_6_) film on a SiO_2_/Si substrate.

The resulting film was deposited over the entire area on a SiO_2_/Si substrate (Fig. 2a). Scanning electron microscopy (SEM) and atomic force microscopy (AFM) images show a surface composed of small crystallites with good uniformity over the substrate (Fig. 2b, c and S5, respectively). AFM analysis of films synthesized with different precursor amounts showed that the film thickness could be controlled in the range of ∼56.9–248.6 nm (Fig. S6 and Table S1). Under the optimized precursor condition, the representative film exhibited a thickness of ∼140 nm and a multigrain surface morphology, with a root-mean-square roughness (*R*_q_) of ∼29.74 nm.

**Fig. 3:**
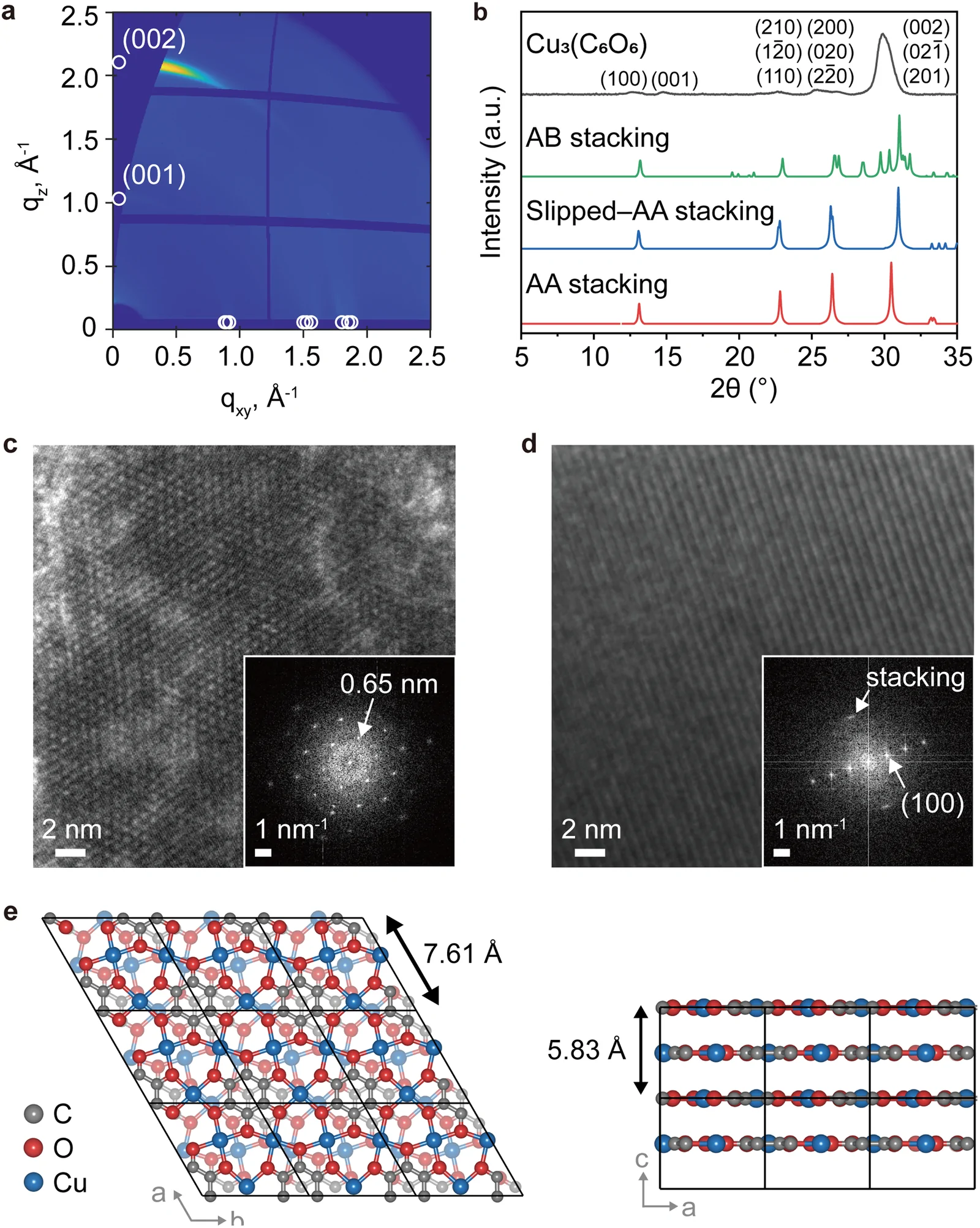
(a) GIWAXS pattern of the Cu_3_(C_6_O_6_) thin film. (b) Crystal structure of AB-stacked Cu_3_(C_6_O_6_) determined by Pawley refinement, shown in top and side views. (c) Simulated diffraction patterns for AA, slipped-AA, and AB stacking modes of Cu_3_(C_6_O_6_), compared with the experimental GIWAXS profile. (d) HRTEM image of the Cu_3_(C_6_O_6_) film showing a honeycomb lattice viewed along the [001] zone axis; the inset FFT indicates a lattice spacing of 0.65 nm. (e) HRTEM image showing stacked layers in the Cu_3_(C_6_O_6_) film; the inset FFT exhibits diffusive arcs corresponding to an interlayer spacing of 0.29 nm.

Grazing-incidence wide-angle X-ray scattering (GIWAXS) pattern of the resulting film showed two peaks around *Q*_z_ = 1.05 and 2.10, assignable to the (001) and (002) planes of a typical hexagonal unit cell in face-on orientation, respectively, indicating that the Cu_3_(C_6_O_6_) film predominantly adopts a face-on orientation (Fig. 3a). In order to determine the crystal structure of the resulting film, Pawley refinement was performed from the integrated intensity profile (Fig. S7). The Pawley refinement result matches well with the experimental data, with *R*_wp_ = 3.70%, *R*_wp_ (without background) = 3.69% and *R*_p_ = 9.08%. The unit cell was optimized to a triclinic unit cell with *a* = 7.83, *b* = 7.61, *c* = 5.83, *α* = 92.0°, *β* = 88.8°, and *γ* = 117.7° (Fig. S8). 2D c-CPs can form various stacking structures depending on their structural stability and synthetic conditions.^29–31^ To determine the stacking order of Cu_3_(C_6_O_6_), various stacking modes of Cu_3_(C_6_O_6_) were simulated and compared (Fig. 3b and S9). Detailed information on the structural optimization process is provided in the SI. In short, among the various possible stacking modes, the AB stacking mode reproduces a series of peaks around 29–31°, which are absent in the slipped-AA mode and can be assigned to a combination of (002), (021̄), and (201) peaks. To further support the stacking analysis, the out-of-plane diffraction profile extracted from the experimental GIWAXS pattern was compared with simulated XRD patterns of (001)-oriented Cu_3_(C_6_O_6_) structures with AA, slipped-AA, and AB stacking (Fig. S10). In the AA-stacked model, the (001) reflection near 15° is absent, whereas slipped-AA and AB stacking give rise to this reflection due to interlayer offset and the doubled periodicity along the *c* direction. The relatively clear (001) reflection in the experimental profile is therefore more consistent with a slipped-AA or AB offset stacking structure than with the AA-stacked model. Moreover, the intensity and position of the experimental (001) reflection show better agreement with the AB-stacked model than with the slipped-AA-stacked model. To further examine the local lattice and stacking structure, transmission electron microscopy (TEM) analysis was performed. TEM images show honeycomb lattices viewed along [001] zone axis, and the corresponding fast Fourier transform (FFT) images identify a lattice parameter of 0.65 nm (Fig. 3c). Furthermore, clearly stacked layers were observed with diffusive arcs at 0.34 Å^−1^, which corresponds to an interlayer stacking distance of 0.29 nm for Cu_3_(C_6_O_6_) in real space (Fig. 3d).^32,33^ These TEM observations are consistent with the layered Cu_3_(C_6_O_6_) structure suggested by GIWAXS analysis.

In addition, selected HRTEM images and the corresponding FFT patterns were further analyzed to compare the experimental local diffraction pattern with simulated stacking models (Fig. S11). The FFT pattern observed along the [111] zone axis contains spots corresponding to the {11̄0} family of reflections as well as the (101̄)/(011̄) spots. In the simulated FFT patterns, the (101̄) and (011̄) spots are absent in the AA-stacked model, weak in the slipped-AA-stacked model, and clearly observed in the AB-stacked model. Therefore, based on the combined GIWAXS and HRTEM/FFT analyses, an AB-stacked layered Cu_3_(C_6_O_6_) structure model was constructed (Fig. 3e), which differs from the previously reported solution-derived AA-stacked model.

The elemental composition of the Cu_3_(C_6_O_6_) film was examined by TEM-EDS and quantitative X-ray photoelectron spectroscopy (XPS) analyses. The Cu : O ratio determined by TEM-EDS was approximately 0.92 : 2 (Fig. S4 and Table S2), while the Cu : O ratio determined by quantitative XPS was approximately 0.89 : 2 (Table S3). These values are close to the ideal CuO_2_ unit composition of Cu : O = 1 : 2, supporting the proposed Cu_3_(C_6_O_6_) stoichiometry.

XPS and Fourier-transform infrared spectroscopy (FT-IR) analyses were used to understand the chemical environment of the Cu_3_(C_6_O_6_) film (Fig. S12 and S13, respectively). The XPS Cu 2p spectrum exhibits mixed oxidation states of Cu^2+^ and Cu^+^, together with satellite peaks associated with Cu^2+^. The C 1s spectrum shows that the C=O: C–O ratio of Cu_3_(C_6_O_6_) is ∼1.12 : 1, which is close to the previously reported value of 1.26 : 1 for a fully coordinated Cu_3_(C_6_O_6_) structure.^26^ This result therefore suggests a similar coordination environment, with minimal contribution from uncoordinated O species. FT-IR spectra revealed that the alkyl C–H signal of Cu(*t*Buacac)_2_ at 2977 cm^−1^, O–H signal at 3359 cm^−1^, and C=O signal at 1613 cm^−1^ completely vanished in the Cu_3_(C_6_O_6_) film. This indicates successful coordination between copper ions and OH groups.

**Fig. 4:**
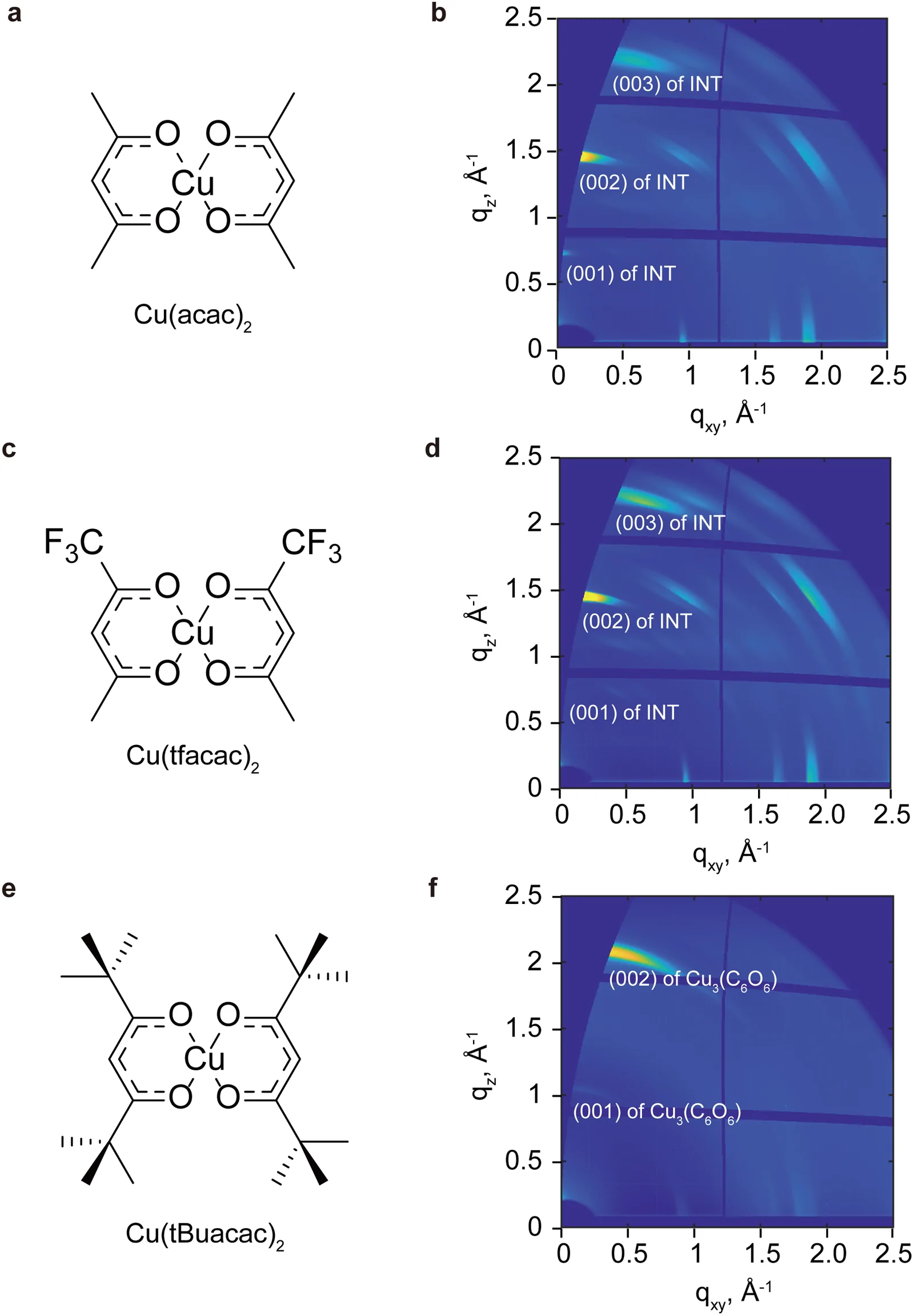
Precursor-dependent film formation using Cu(acac)_2_, Cu(tfacac)_2_, and Cu(*t*Buacac)_2_. (a, c and e) Chemical structures of (a) Cu(acac)_2_ (acac = acetylacetonate), (c) Cu(tfacac)_2_ (tfacac = trifluoroacetylacetonate), and (e) Cu(*t*Buacac)_2_ (*t*Buacac = *tert*-butylacetoacetate). (b, d and f) GIWAXS patterns of the resulting films grown from (b) Cu(acac)_2_, (d) Cu(tfacac)_2_, and (f) Cu(*t*Buacac)_2_.

As summarized in Table S4, most reported single-step CVD syntheses of M_3_(C_6_X_6_)_n_ films (X = O or S; *n* = 1 or 2) have relied on M(acac)_*n*_ metal precursors. To clarify the role of Cu(*t*Buacac)_2_ as a decisive precursor in directing selective formation of a 2D c-CP rather than a 3D MOF, control experiments were performed using Cu(acac)_2_, Cu(tfacac)_2_ (tfacac = trifluoroacetylacetonate), and Cu(*t*Buacac)_2_ (Fig. 4a–f). As described above, INT refers to an intermediate containing kagome-structured layers sandwiched by THQ pillars between the layers. Cu(acac)_2_ and Cu(tfacac)_2_ produced the INT phase, whereas Cu(*t*Buacac)_2_ led to the formation of Cu_3_(C_6_O_6_) structure. This comparison indicates that the greater steric bulkiness of the *t*Buacac ligand plays a key role in suppressing THQ pillar coordination and directing the formation of the layered 2D c-CP structure.

**Fig. 5:**
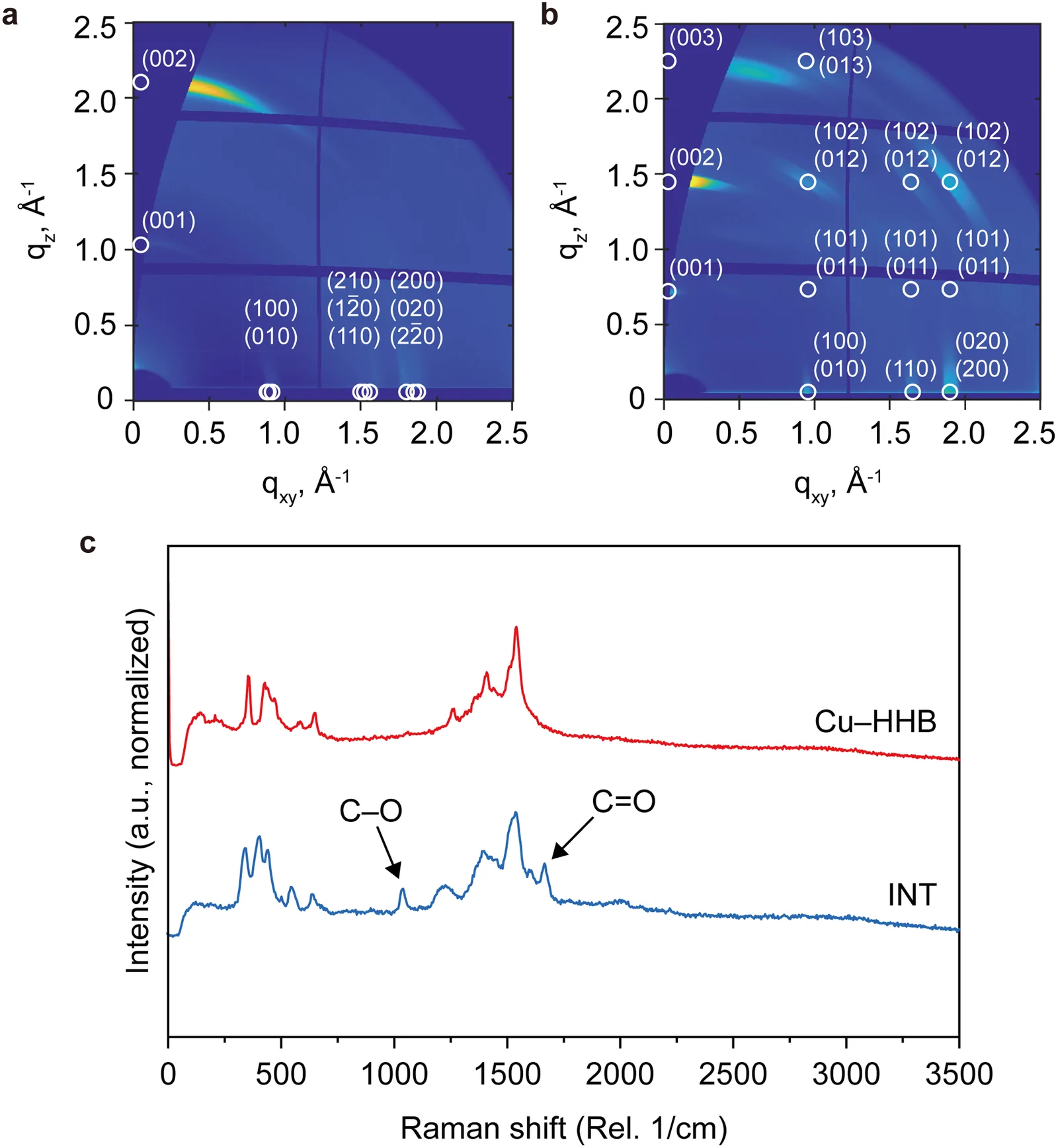
(a) GIWAXS pattern of the Cu_3_(C_6_O_6_) thin film grown from Cu(*t*Buacac)_2_. (b) GIWAXS pattern of the INT film grown from Cu(acac)_2_. (c) Raman spectra of the Cu_3_(C_6_O_6_) thin film and INT.

The structural difference between the Cu(*t*Buacac)_2_-derived Cu_3_(C_6_O_6_) film and the Cu(acac)_2_-derived INT film was then examined in more detail by GIWAXS analysis (Fig. 5a and b). Both films exhibit in-plane diffraction peaks corresponding to the (100), (210), and (200) planes, which imply the formation of hexagonal lattices in the ab plane. However, the INT film exhibits a larger periodicity along the out-of-plane direction (8.4 Å) than Cu_3_(C_6_O_6_) (5.8 Å). This difference in structural selectivity during the CVD reaction is attributed to the greater steric bulkiness of the *t*Buacac ligand relative to the acac ligand. This interpretation is supported by the reported transformation mechanism of INT to an edge-on Cu_3_(C_6_O_6_)_2_ MOF,^27^ in which the 3D MOF, INT, forms initially and transforms into the 2D MOF Cu_3_(C_6_O_6_)_2_ upon annealing. The chemical difference between Cu_3_(C_6_O_6_) and INT was also examined by Raman spectroscopy (Fig. 5c and S14). Unlike INT, the Raman spectrum of Cu_3_(C_6_O_6_) does not exhibit distinctive peaks of 1054 and 1669 cm^−1^, which represent the uncoordinated C–O and C=O stretching modes of the pillar THQ.^34^ This absence is consistent with pillar removal and supports the formation of the layered 2D c-CP structure.

The growth process under the Cu(*t*Buacac)_2_ condition was further examined by time-dependent *ex situ* GIWAXS analysis using films quenched after reaction times of 1, 3, 5, 10, and 30 min under identical CVD conditions (Fig. S15). The characteristic (001) and (002) reflections of INT were not observed at any examined reaction time. Instead, diffraction peaks corresponding to Cu_3_(C_6_O_6_) gradually developed, with in-plane reflections becoming visible after 10 min and a well-defined Cu_3_(C_6_O_6_) pattern obtained after 30 min. Taken together, these results support the interpretation that the bulky ligand in the Cu(*t*Buacac)_2_ metal precursor suppresses axial THQ coordination and pillar formation while allowing in-plane Cu–O coordination during the reversible surface reaction. Accordingly, when Cu(*t*Buacac)_2_ is used, the formation of the INT phase is suppressed, and a face-on Cu_3_(C_6_O_6_) film is formed directly.

**Fig. 6:**
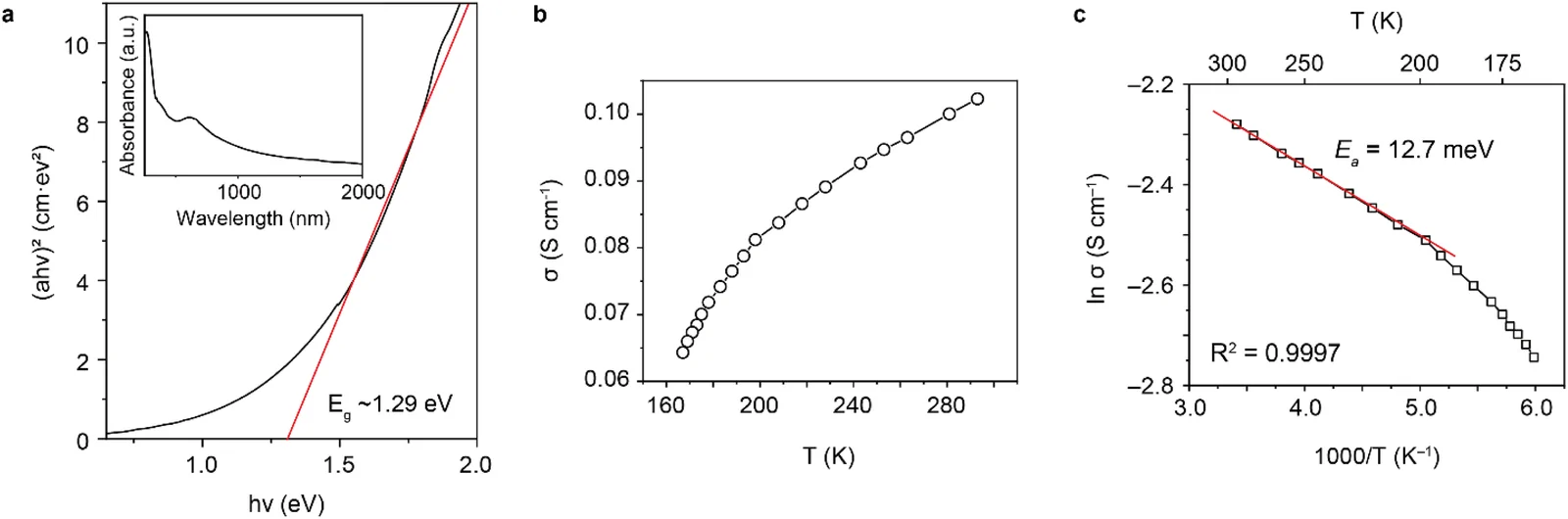
Optical and temperature-dependent electrical properties of the Cu_3_(C_6_O_6_) film. (a) Tauc plot obtained from the UV-vis-NIR absorption spectrum. The inset shows the corresponding absorption spectrum. (b) Temperature-dependent conductivity of the Cu_3_(C_6_O_6_) film. (c) Arrhenius plot of ln *σ versus* 1000/*T*. The apparent activation energy was extracted from the linear fit in the 200–300 K range.

Ultraviolet-visible-near infrared (UV-vis-NIR) spectra were obtained on quartz substrates to estimate the optical bandgap of Cu_3_(C_6_O_6_) (Fig. 6a inset), and the Tauc plot gives an optical bandgap of ∼1.29 eV (Fig. 6a), which is similar to the reported value for Cu_3_(C_6_O_6_) powder.^35^

The electrical conductivity of the Cu_3_(C_6_O_6_) film was then examined using a four-probe configuration. Linear *I*–*V* characteristics were observed for the measured film, indicating ohmic contact formation (Fig. S16). The room-temperature conductivity was approximately 0.10 S cm^−1^, which is about 4.3 times higher than that of the previously reported pelletized Cu_3_(C_6_O_6_) powder, 0.026 S cm^−1^. Temperature-dependent conductivity measurements showed that the conductivity decreases upon cooling, indicating thermally assisted transport in the measured temperature range (Fig. 6b). Arrhenius analysis gave an apparent activation energy of 12.7 meV from the linear region between 200 and 300 K (Fig. 6c). This activation energy is much smaller than the high-temperature activation energy reported for pelletized Cu_3_(C_6_O_6_) powder and is comparable to the value reported for the lower-temperature region.^35^ These results indicate that the temperature-dependent conductivity reflects a low-energy transport barrier in the film rather than direct excitation across the intrinsic optical bandgap.

The measured conductivity of the film can also be discussed in terms of its stacking structure. Previous calculations on Cu_3_(C_6_O_6_) suggested that charge transport along the interlayer direction, that is, the *c*-axis pathway, is more favorable than transport within the 2D plane, indicating that interlayer orbital overlap is an important transport pathway.^35^ In addition, computational studies of π-stacked 2D porous framework materials showed that larger layer offsets can reduce interlayer electronic coupling and increase the effective mass along the stacking direction, leading to less efficient vertical charge transport.^36,37^ In this context, the offset stacking suggested by the AB-stacked model of the present Cu_3_(C_6_O_6_) film may limit the interlayer transport pathway, which may contribute to its measured conductivity being moderately higher than that of the previously reported pelletized powder sample.

Finally, the ambient stability of the Cu_3_(C_6_O_6_) film was evaluated by electrical and GIWAXS measurements (Fig. S16). The film retained 97.3% of its initial conductivity after 2 weeks in air, and all characteristic crystalline reflections of Cu_3_(C_6_O_6_) remained clearly observable after 1 year of ambient storage. These results indicate that both the electrical characteristics and crystalline structure of the Cu_3_(C_6_O_6_) film are largely maintained under ambient conditions.

## Conclusions

In summary, a 2D kagome Cu_3_(C_6_O_6_) c-CP thin film was fabricated by CVD. Use of Cu(*t*Buacac)_2_ instead of Cu(acac)_2_ redirected the vapor-phase coordination pathway by suppressing axial THQ coordination, thereby enabling direct formation of a layered 2D c-CP structure. The resulting Cu_3_(C_6_O_6_) thin film is further suggested to have an offset-stacked layered arrangement, which differs from the AA-stacked model reported for the solution-derived structure. This indicates that the vapor-phase growth environment can influence not only the framework formation but also the layer arrangement. These findings demonstrate that metal precursors not only work as simple metal sources but also affect the coordination environment. More broadly, this work provides a useful foundation for future efforts to design precursors for targeted c-CP synthesis under CVD conditions, where precursor selection inevitably plays a decisive role in determining the accessible coordination geometry and structural outcome.

## Conflicts of interest

There are no conflicts to declare.

## Supplementary Material

RA-016-D6RA05487K-s001

## Data Availability

The data that support the findings of this study are available from the corresponding authors upon reasonable request. The data supporting this article have been included as part of the supplementary information (SI). Supplementary information is available. See DOI: https://doi.org/10.1039/d6ra05487k.
