# Supplementary material for: Direct synthesis of two-dimensional Cu3(C6O6) kagome conjugated coordination polymer thin films via chemical vapor deposition
Source: RSC Adv. 2026 Jul 22;16(40):45834–9. doi: 10.1039/d6ra05487k (PMC13390115; doi:10.1039/d6ra05487k)
Supplement: Supplementary file 1 [file RA-016-D6RA05487K-s001.pdf]

Supplementary Information (SI) for RSC Advances.
This journal is © The Royal Society of Chemistry 2026

Supplementary information

Direct Synthesis of Two-Dimensional $Cu_3(C_6O_6)$ Kagome Conjugated Coordination Polymer Thin Films via Chemical Vapor Deposition

Hyeonwoo Lee[a] and Hee Cheul Choi[a]*

[a]*Department of Chemistry, Pohang University of Science and Technology (POSTECH), Pohang 37673, Republic of Korea*

Corresponding author: Hee Cheul Choi – Email: choihc@postech.edu

Table of contents ***page***

Experimental methods S3

Materials and general information S3

Synthesis of $Cu_3(C_6O_6)$ via chemical vapor deposition (CVD) process S3

Transmission electron microscopy (TEM) S3

Grazing-incidence wide-angle X-ray scattering (GIWAXS) S3

X-ray photoelectron spectroscopy (XPS) S4

Fourier transform infrared spectroscopy (FT-IR) S4

Device fabrication and measurement S4

Computational details S4

Fig. S1 Digital photograph of the CVD system setup S5

Fig. S2 Optical image of $Cu_3(C_6O_6)$ film S5

Fig. S3 SEM-EDS image of $Cu_3(C_6O_6)$ film S6

Fig. S4 TEM-EDS image of $Cu_3(C_6O_6)$ film S6

Fig. S5 Uniformity analysis of $Cu_3(C_6O_6)$ film S7

Fig. S6 AFM image and height profile of $Cu_3(C_6O_6)$ film S8

Table S1. Thickness of synthesized $Cu_3(C_6O_6)$ films S8

Fig. S7 Detailed GIWAXS intensity profile analysis S9

Fig. S8 Pawley refinement plot S10

Fig. S9 Simulated interlayer stacking structure of $Cu_3(C_6O_6)$ film S11

Fig. S10 Out-of-plane diffraction analysis of $Cu_3(C_6O_6)$ film S12

Fig. S11 HRTEM analysis of $Cu_3(C_6O_6)$ film S13

Table S2. Quantitative TEM-EDS analysis of $Cu_3(C_6O_6)$ S14

Table S3. Quantitative XPS analysis of $Cu_3(C_6O_6)$ film S14

Fig. S12 Deconvoluted XPS spectra of $Cu_3(C_6O_6)$ film S15

Fig. S13 ATR FT-IR spectra of $Cu_3(C_6O_6)$ film S16

Fig. S14 Raman spectra of $Cu_3(C_6O_6)$ film S17

Fig. S15 Time-dependent ex situ GIWAXS analysis of $Cu_3(C_6O_6)$ film S18

Fig. S16 Stability of $Cu_3(C_6O_6)$ film under ambient conditions S19

Table S4. Summary of reported single-step CVD syntheses of coordination polymer thin films S20

References S22

Experimental methods

Materials and general information

Copper(II) tert-butylacetoacetate ($Cu(tBuacac)_2$) ($C_{16}H_{26}CuO_6$, > 97.0 % (T), Sigma Aldrich) and tetrahydroxy-1,4-benzoquinone hydrate (THQ) ($C_6H_4O_6 \cdot xH_2O$, > 96.0% (T), TCI) were purchased from commercial sources and used without further purification. A 300 nm SiO_2/Si substrate (Nanopia Co., Ltd.) was cut into 1 cm × 1 cm pieces and used as the growth substrate. Ar (99.999%, Donghae Gas Ind. Co., Ltd.) was used as the carrier gas. $Cu_3(C_6O_6)$ and intermediate (INT) films were synthesized in a tube-type furnace (Lindberg/Blue M, TF55030C-1). Quartz tubes (25 mm outer diameter × 1.5 mm wall thickness × 600 mm length; Dawon Materials Science) were used without further treatment. Test tubes (10 mm outer diameter × 100 mm length; Daihan Scientific Co., Ltd.) were placed inside the quartz tube to construct a face-to-face inner-tube configuration. Grazing-incidence wide-angle X-ray scattering (GIWAXS) patterns were acquired at the 3C beamline of the Pohang Accelerator Laboratory (Pohang, Korea) using synchrotron radiation. The collected data were converted into q values or 2θ values referenced to Cu Kα radiation (λ = 1.54056 Å).

Synthesis of $Cu_3(C_6O_6)$ film via chemical vapor deposition (CVD) process

$Cu_3(C_6O_6)$ thin films were synthesized using a chemical vapor deposition (CVD) method in a tube–type furnace (Lindberg/Blue M, model TF55030C–1), based on the reported face-to-face inner tube CVD system.[1] $Cu(tBuacac)_2$ and THQ were used as the precursors, with 6 mg of $Cu(tBuacac)_2$ and 2 mg of THQ loaded separately, corresponding to a fixed mass ratio of 3:1. Each precursor was loaded into a separate test tube, and the test tubes were placed inside a quartz tube installed in the furnace. The $Cu(tBuacac)_2$-containing test tube was located 14.5 cm upstream from the furnace center, while the THQ–containing test tube was located 10.5 cm downstream. SiO_2/Si substrates, diced into 1.0 cm × 1.0 cm pieces, were cleaned by sequential rinsing in deionized water, acetone, and isopropyl alcohol for 5 min each, then introduced into the quartz tube. For film collection, the substrate center was aligned 1.5 cm downstream from the furnace center. Before heating, the quartz tube was evacuated to 10 mTorr and subsequently purged with argon gas at 100 sccm for 10 min. The argon flow was then reduced to 20 sccm, giving an internal pressure of approximately 140 mTorr. The furnace was heated to 140 °C at a ramping rate of 30 °C min^{-1} and held at that temperature for 30 min to promote film growth. After completion of the reaction, the furnace power was shut off and the lid was opened, allowing the system to cool naturally to room temperature. The resulting $Cu_3(C_6O_6)$ thin films on SiO_2/Si substrates were then collected for subsequent characterization and device fabrication. For TEM analysis, powder samples were obtained by physically scraping the product films.

Transmission electron microscopy (TEM)

TEM data were collected using a JEM-2200FS microscope (JEOL, 200 kV) equipped with an image Cs corrector at the National Institute for Nanomaterials Technology (NINT). Data analysis was carried out using Gatan DigitalMicrograph software.

Grazing-incidence wide-angle X-ray scattering (GIWAXS)

GIWAXS data were acquired at the 3C beamline of the Pohang Accelerator Laboratory (PAL) with a photon

energy of 11.3 keV and an energy resolution ($\Delta E/E$) of ~2×10^{-4}. The beamline was equipped with a DCM Si(111) crystal monochromator and an Eiger X4M detector, and provided a beam size of 60 (V) × 600 (H) μm^2. Each dataset was calibrated using silver behenate (AgBeh) as a standard material with the beamline-specific software PGIXS. GIWAXS pattern simulation and Miller index assignment for the experimental patterns were carried out using an publicly available online tool (https://github.com/naveenv92/xray-scattering-tools). This method was based on the procedure described by N. R. Venkatesan et al.

X-ray photoelectron spectroscopy (XPS)

XPS data were collected with a VG SCIENTIFIC ESCALAB 205 spectrometer at the Research Institute of Industrial Science & Technology (RIST). Peak fitting was performed using Origin 2018 software with Gaussian–Lorentzian profiles.

Fourier transform infrared spectroscopy (FT-IR)

Attenuated total reflectance Fourier-transform infrared (ATR FT-IR) spectra were acquired using a Vertex 80/v spectrometer at the 12D beamline of the Pohang Accelerator Laboratory (PAL), equipped with an ATR three-reflection plate with a ZnSe crystal. Data analysis were proceeded with Origin 2018 software.

Device fabrication and measurement

Top-contact four-probe devices were fabricated by depositing Cr (15 nm)/Au (50 nm) electrodes onto the $Cu_3(C_6O_6)$ film through a shadow mask using an e-beam metal evaporator. The channel length and width were 50 and 900 μm, respectively, and each channel was extended to 1000 × 1000 μm^2 contact pads for electrical probing. Electrical measurements on the thin films, including room-temperature conductivity and temperature-dependent conductivity measurements, were carried out in a four-probe configuration using a semiconductor analyzer (Keithley 4200).

Computational details

The $Cu_3(C_6O_6)$ structure was modeled on the basis of the previously reported $Cu_3(C_6O_6)$ structure. The stacking of the $Cu_3(C_6O_6)$ coordination polymer was systematically varied to construct AA, slipped-AA, and AB-stacked models. Structural optimization of each model was performed using the GGA-PBEsol functional with the DND basis set in the $DMol^3$ module of the Materials Studio program. The final lattice parameters of AB-stacked $Cu_3(C_6O_6)$ were determined by Pawley refinement of the experimental GIWAXS integrated profile using the Reflex and $DMol^3$ modules in Materials Studio. While this approach does not directly capture bulk structural information to the same extent as conventional XRD, it was adopted as an alternative because conventional XRD measurements were difficult to obtain from the thin-film sample.

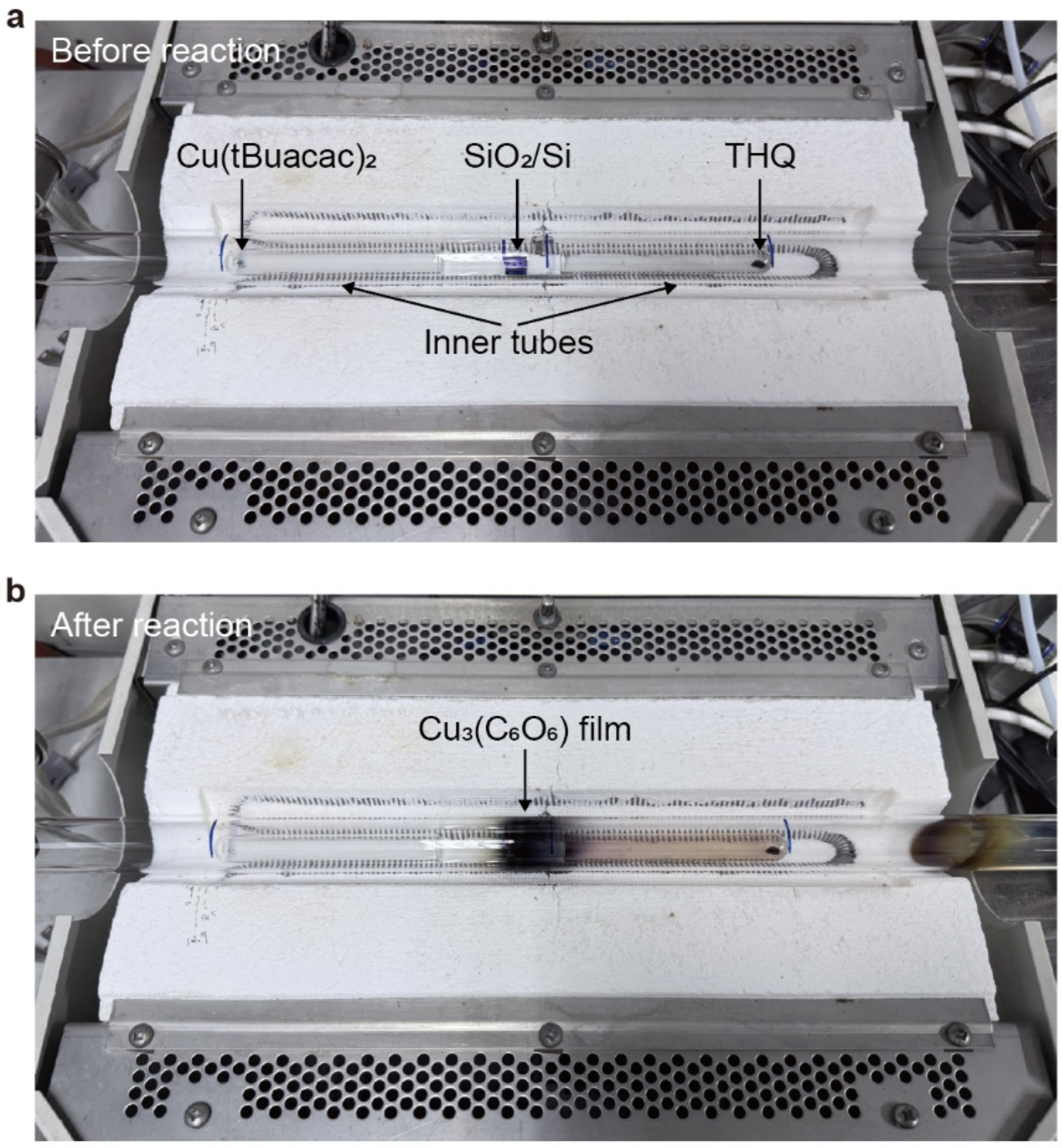

Fig. S1. Digital photographs of the CVD system setup for $Cu_3(C_6O_6)$ film synthesis. (a) Setup before the reaction, showing the positions of the $Cu(tBuacac)_2$ precursor, SiO_2/Si substrate, and THQ precursor inside the face-to-face inner-tube configuration. (b) Setup after the reaction, showing the deposited $Cu_3(C_6O_6)$ film on the substrate.

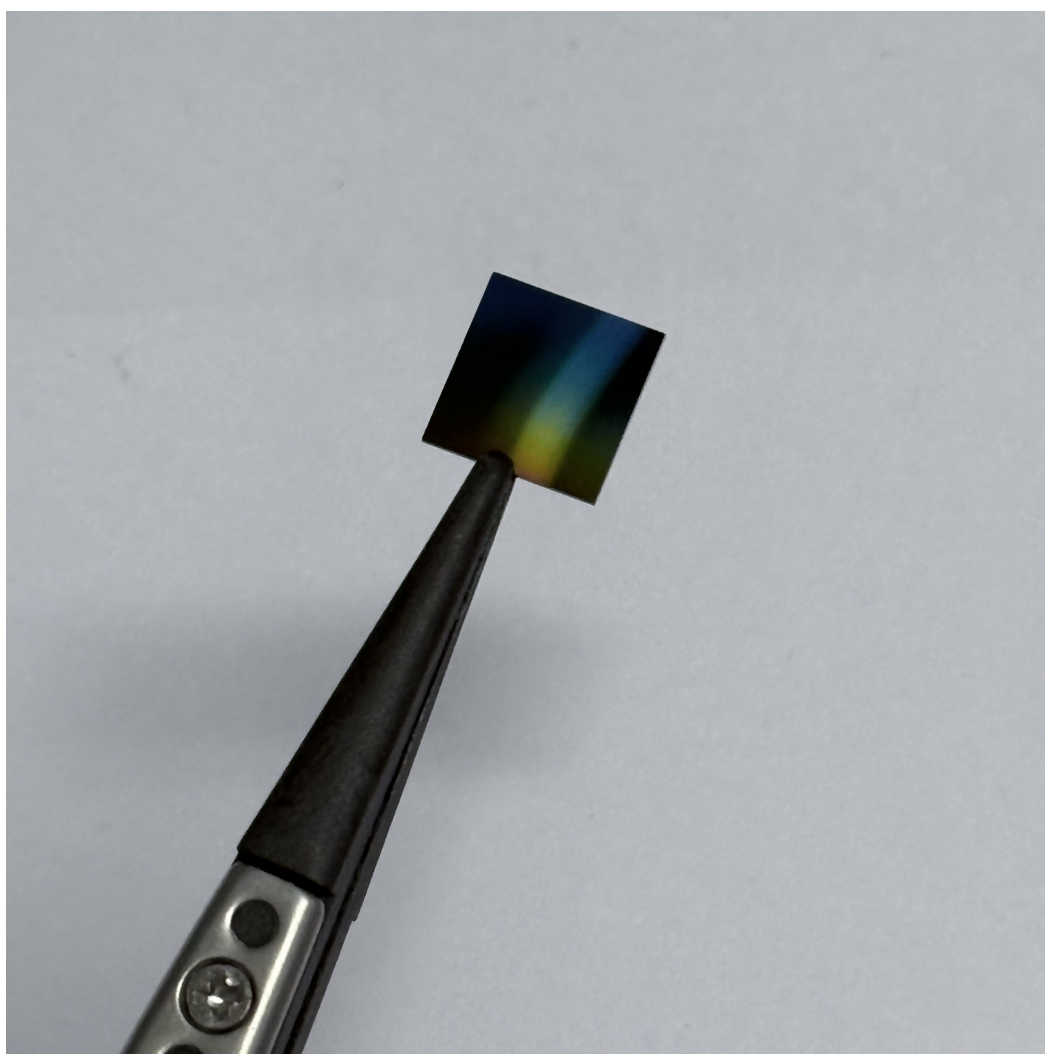

Fig. S2. Optical image of $Cu_3(C_6O_6)$ film, showing high reflectivity.

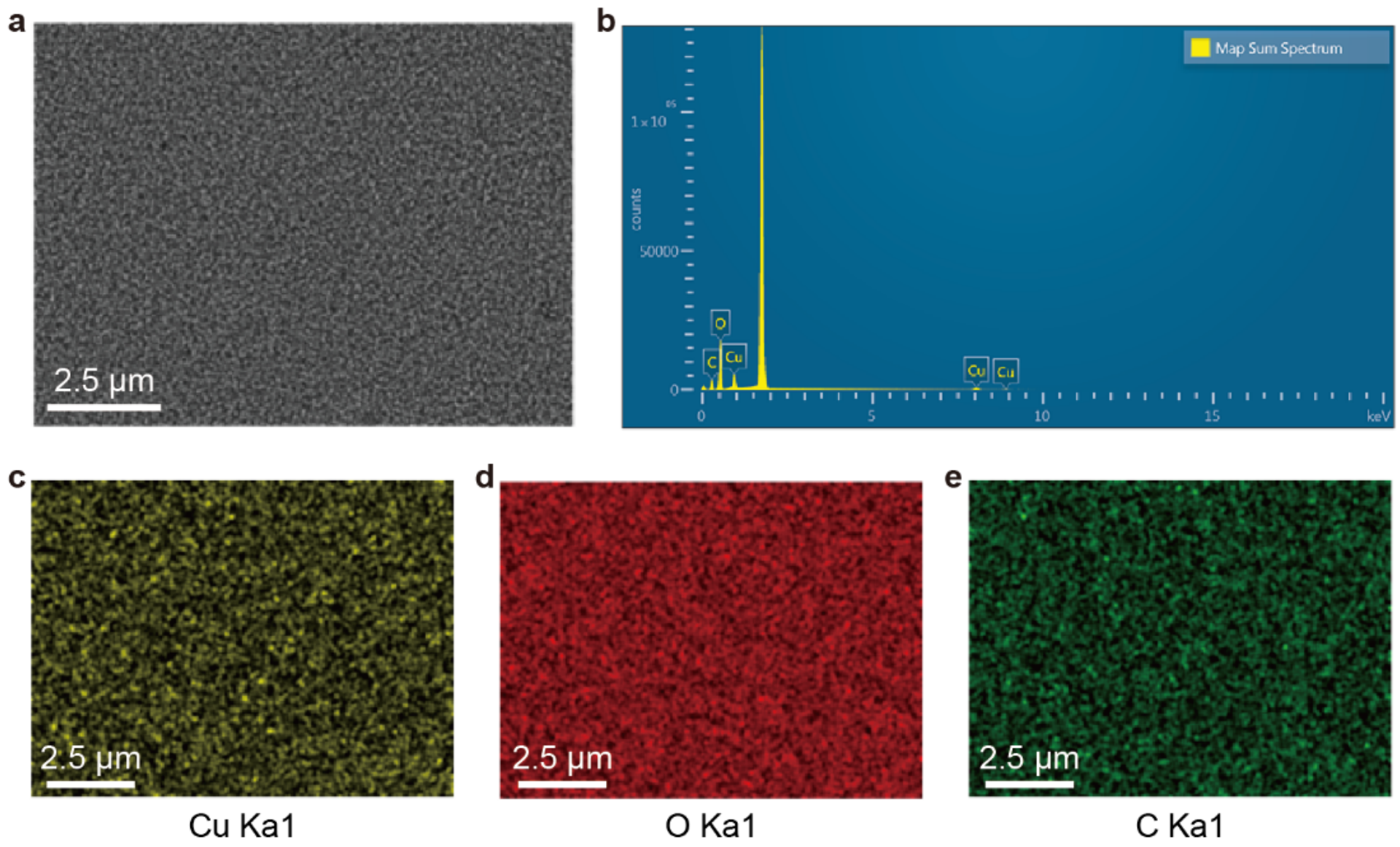

Fig. S3. (a) Representative surface SEM image of $Cu_3(C_6O_6)$ film on SiO_2/Si substrate. (b) Corresponding EDS spectrum for the $Cu_3(C_6O_6)$ film on SiO_2/Si substrate. Elemental mapping images of (c) Cu distribution, (d) O distribution, and (e) C distribution.

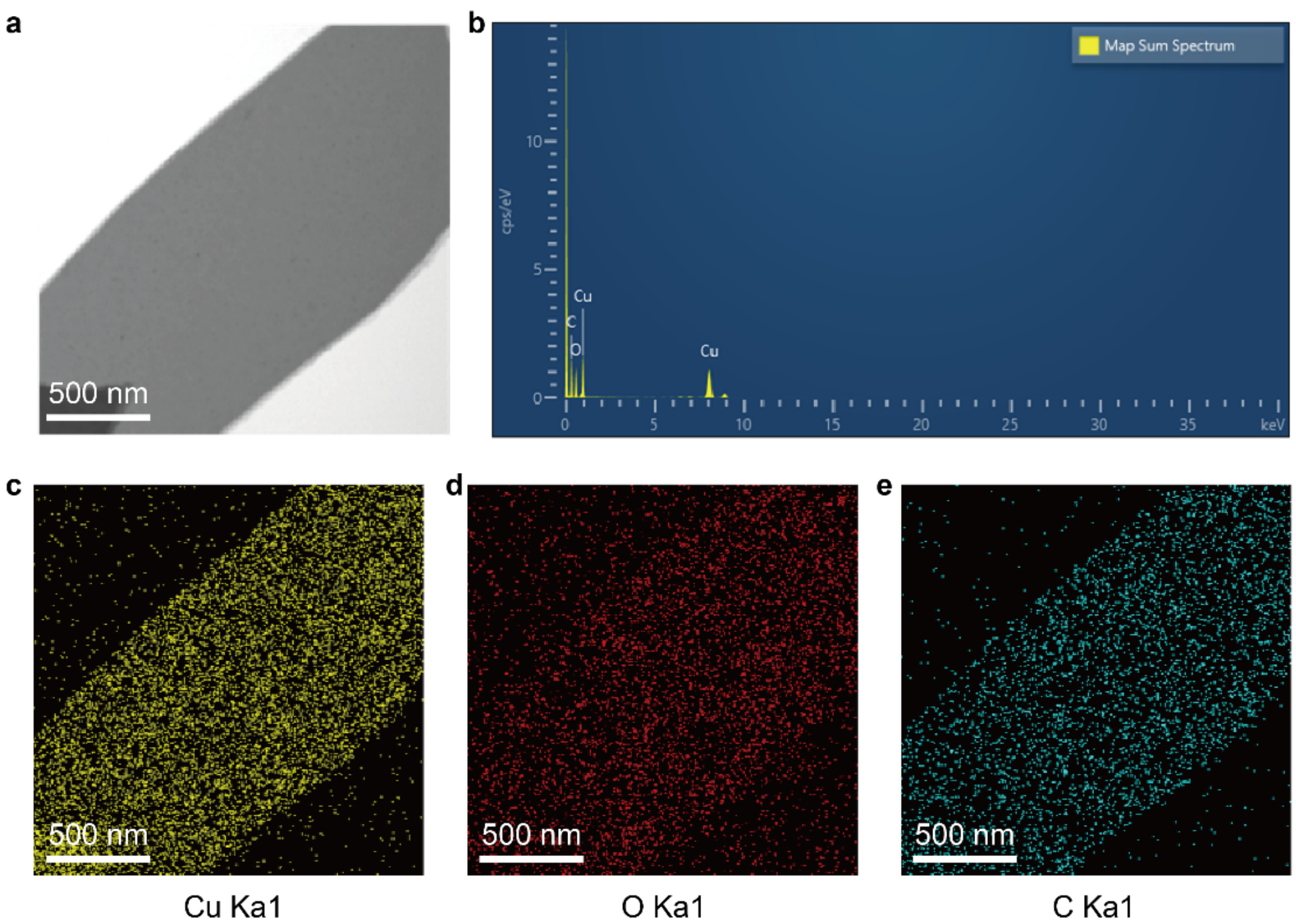

Fig. S4. TEM-EDS analysis of the $Cu_3(C_6O_6)$ film. (a) Representative TEM image of the $Cu_3(C_6O_6)$ film. (b) Corresponding EDS spectrum. Elemental mapping images of (c) Cu distribution, (d) O distribution, and (e) C distribution.

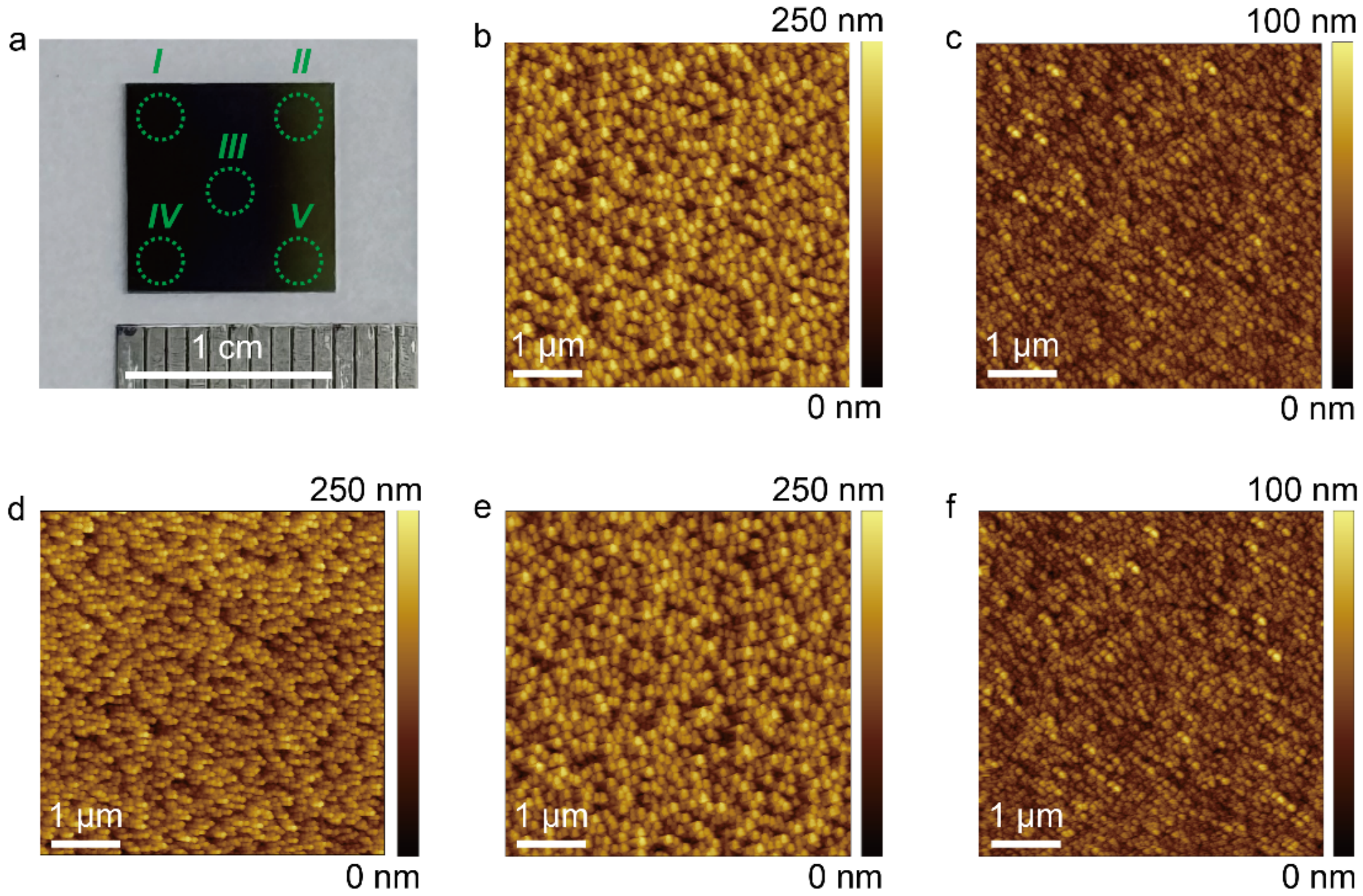

Fig. S5. AFM analysis of film uniformity across a 1 × 1 cm SiO_2/Si substrate. (a) Photograph of the $Cu_3(C_6O_6)$ film with five marked regions. (b–f) AFM topographic images obtained from regions I–V marked in panel (a), respectively. The R_q values of regions I–V are 30.12, 17.45, 29.74, 30.69, and 16.96 nm, respectively.

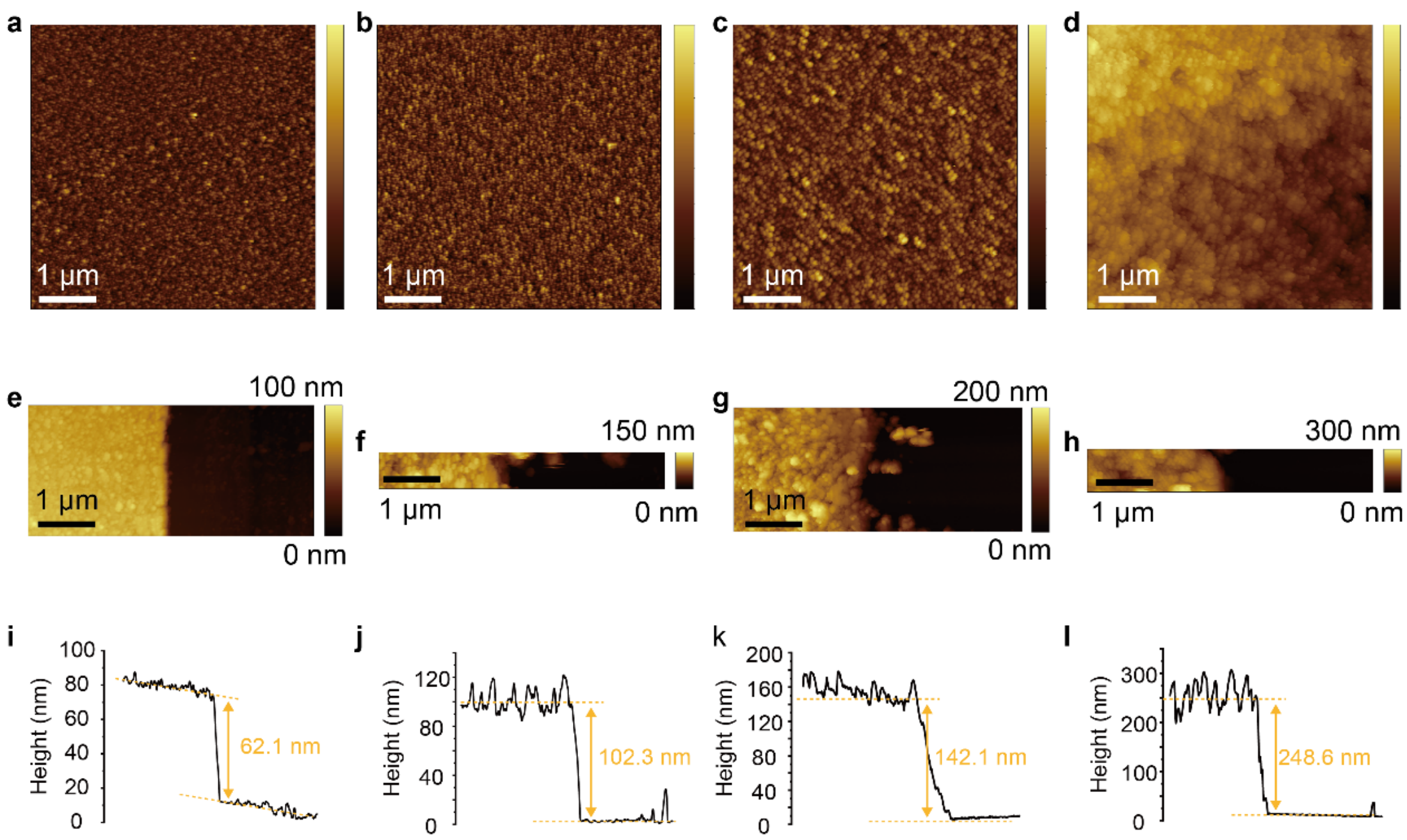

Fig. S6. AFM characterization of $Cu_3(C_6O_6)$ films synthesized with different precursor amounts. (a–d) AFM topographic images, (e–h) scratched-edge AFM images, and (i–l) corresponding height profiles. (a,e,i) 62.1 nm-thick film with R_q of 6.194 nm; (b,f,j) 102.3 nm-thick film with R_q of 18.59 nm; (c,g,k) 142.1 nm-thick film with R_q of 29.74 nm; (d,h,l) 248.6 nm-thick film with R_q of 51.65 nm.

Table S1. Thickness of synthesized $Cu_3(C_6O_6)$ thin films.

$Cu(tBuacac)_2$ (mg)		1.5	3.0	6.0	12.0
THQ (mg)		0.5	1.0	2.0	4.0
Film thickness (nm)	Sample 1	62.1	102.3	142.1	248.6
	Sample 2	57.4	98.1	154.5	265.6
	Sample 3	56.9	107.5	148.2	231.7
	Average[a]	58.8	102.6	148.2	248.6
	SD (nm)[b]	2.3	3.8	5.1	13.8

[a]Average thickness value of the sample 1,2, and 3.

[b]Standard deviation

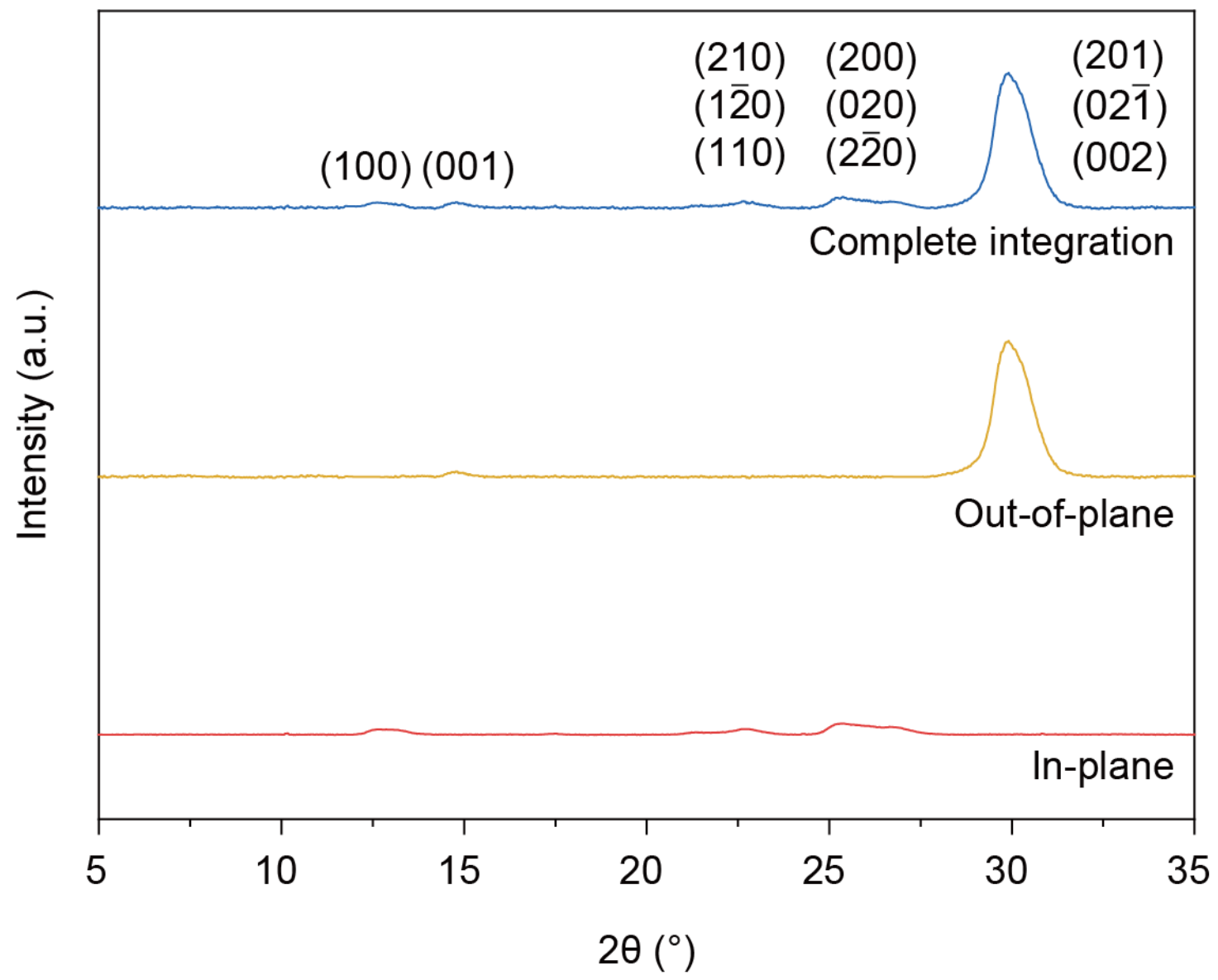

Fig. S7. Integrated GIWAXS profiles of $Cu_3(C_6O_6)$, showing the complete integration, out-of-plane, and in-plane components. Indexed reflections are labeled.

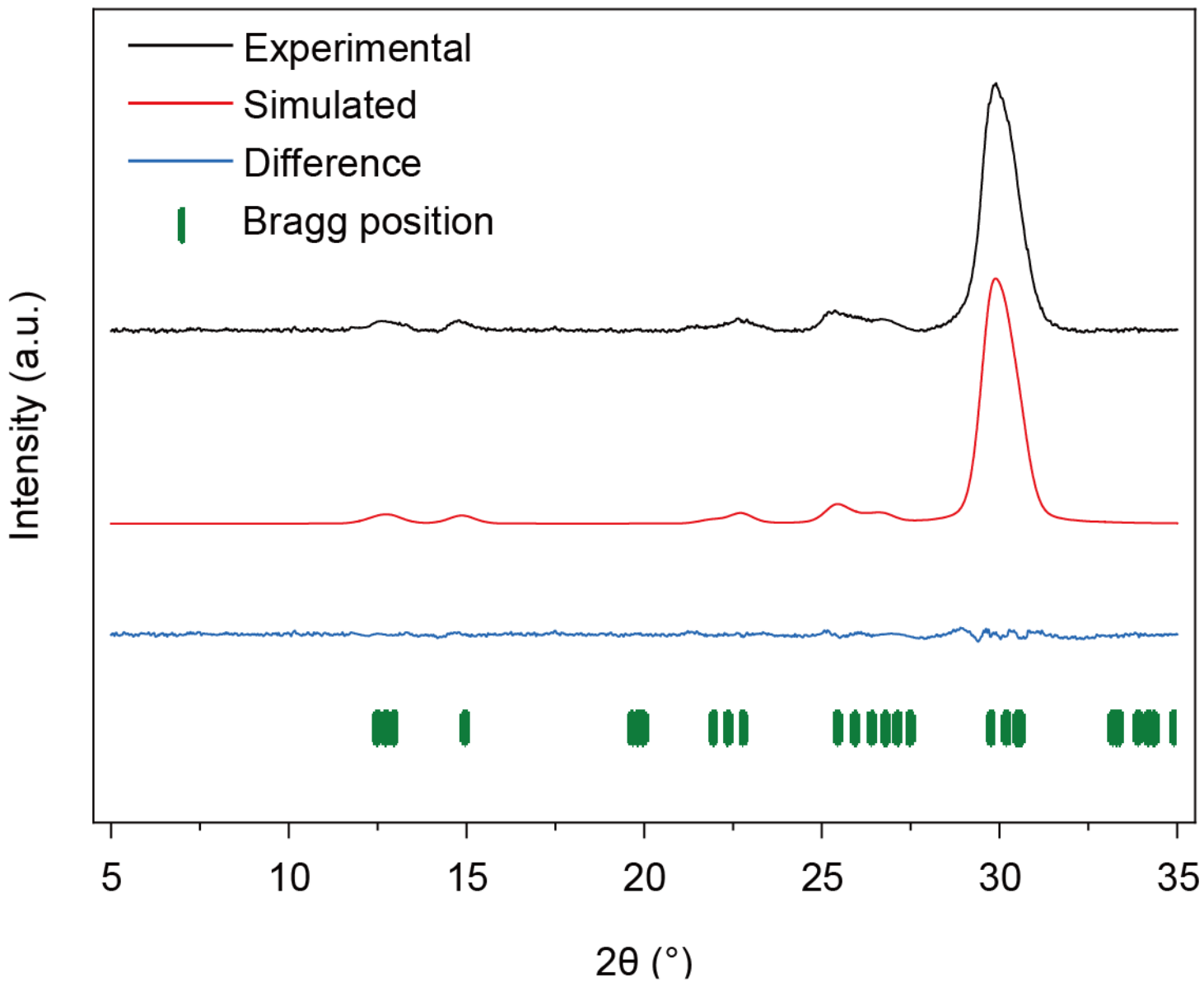

Fig. S8. Pawley refinement plot of the GIWAXS integration profile for the $Cu_3(C_6O_6)$ (R_{wp} = 3.70%, R_{wp} (without background) = 3.69%, R_p = 9.08%).

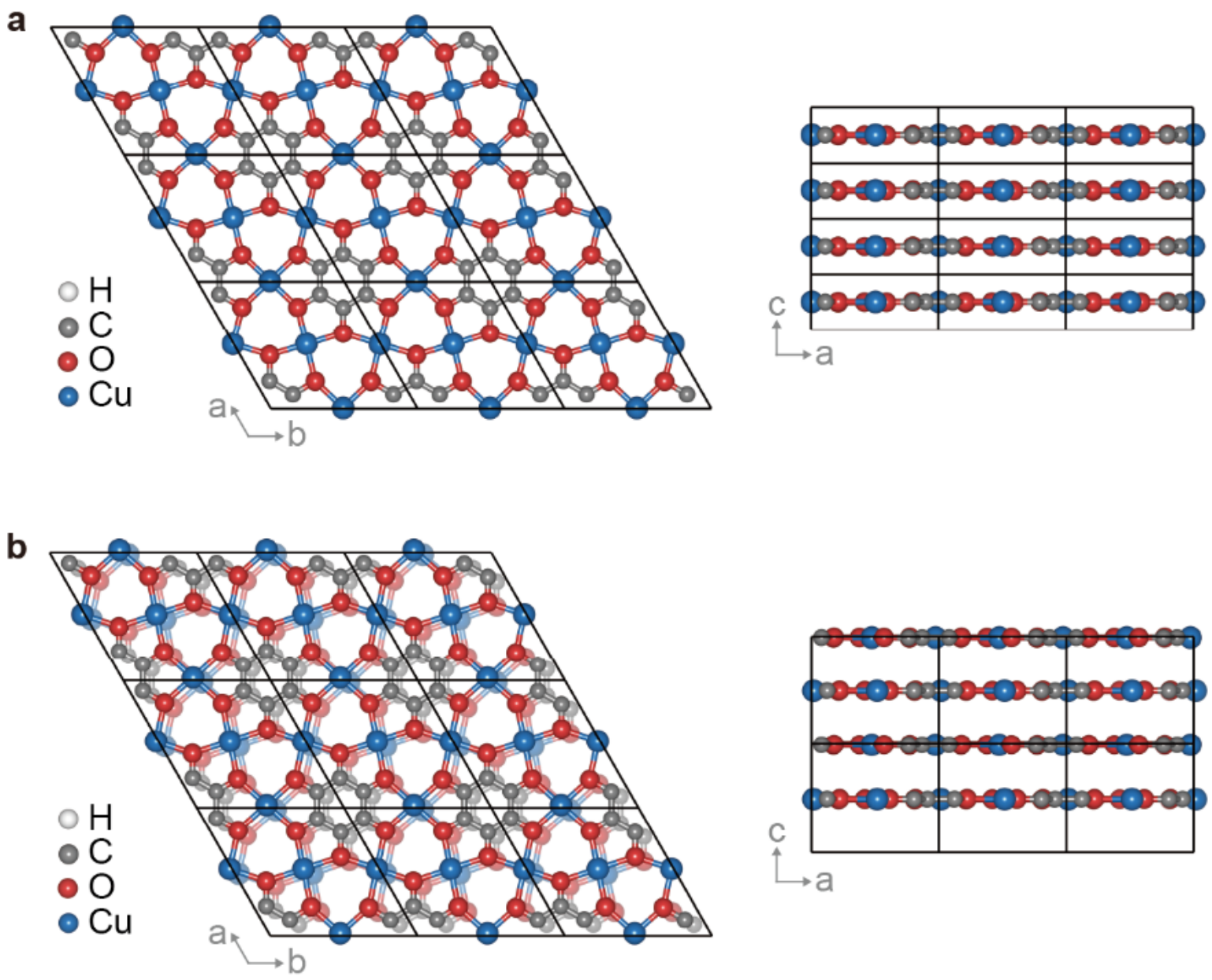

Fig. S9. Simulated interlayer stacking structures of $Cu_3(C_6O_6)$ used for calculation of the diffraction patterns: (a) AA stacking and (b) slipped-AA stacking. Top and side views are shown for each model.[2–4]

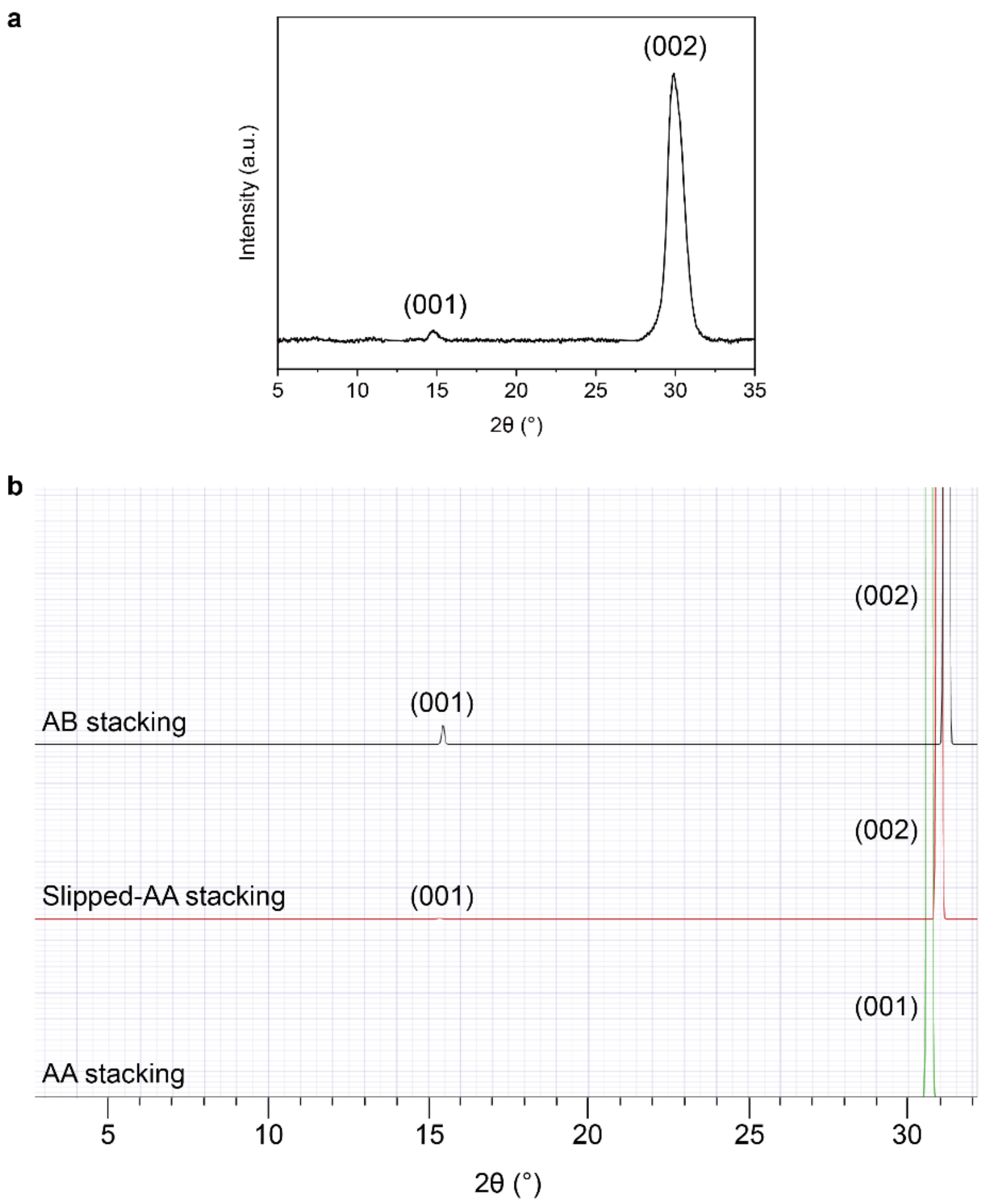

Fig. S10. Out-of-plane GIWAXS analysis and simulated XRD patterns of $Cu_3(C_6O_6)$. (a) Out-of-plane diffraction profile extracted from the experimental GIWAXS pattern. (b) Simulated XRD patterns of (001)-oriented $Cu_3(C_6O_6)$ structures with AB, slipped-AA, and AA stacking.

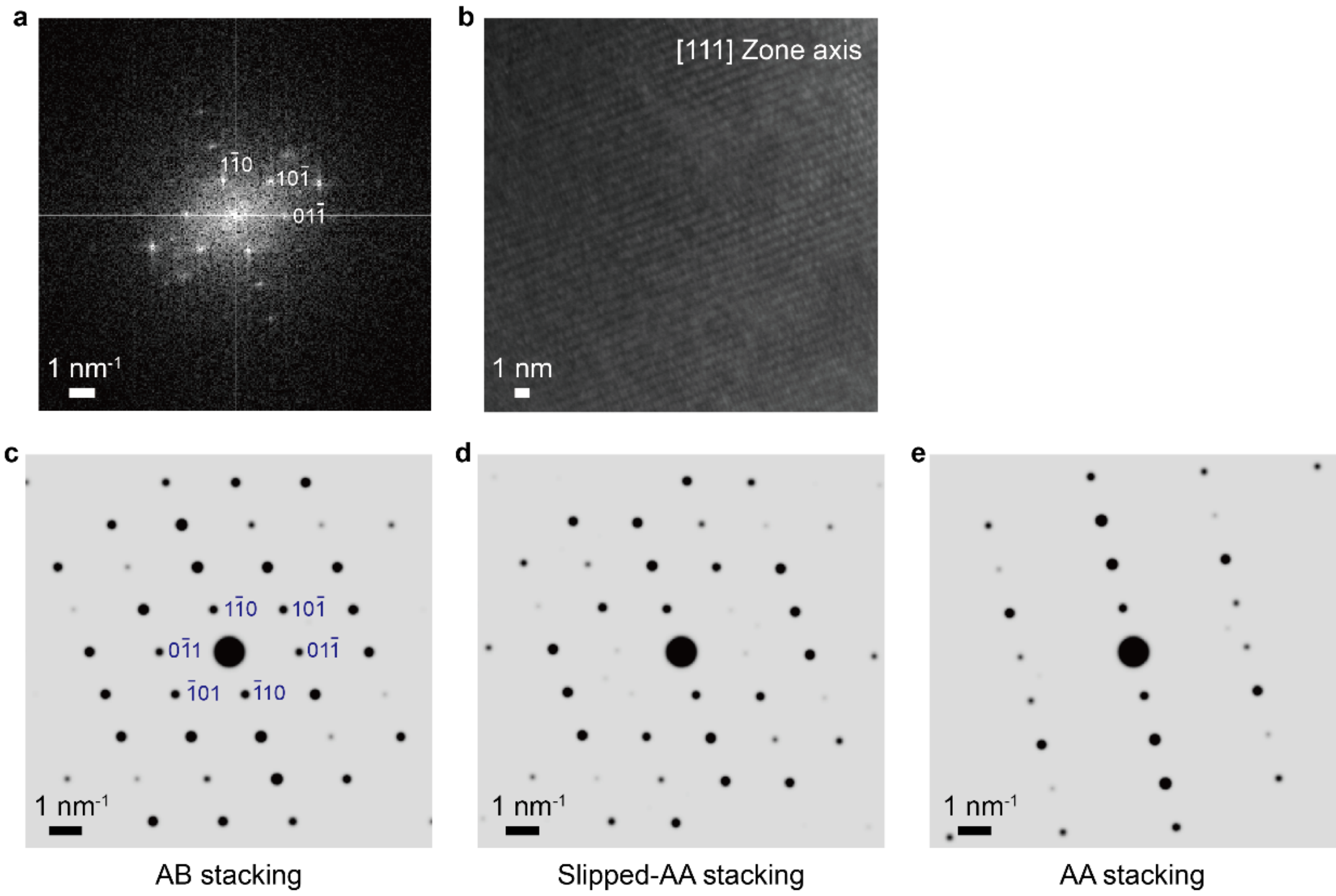

Fig. S11. HRTEM and FFT analysis of $Cu_3(C_6O_6)$. (a) Experimental FFT pattern and (b) HRTEM image observed along the [111] zone axis. Simulated FFT patterns of $Cu_3(C_6O_6)$ with (c) AB stacking, (d) slipped-AA stacking, and (e) AA stacking.

Table S2. Quantitative TEM-EDS weight composition of the $Cu_3(C_6O_6)$ film compared with the theoretical weight composition of $Cu_3(C_6O_6)$. *Note: Because a carbon-coated TEM grid was used, the TEM-EDS data were used only to evaluate the Cu-to-O ratio, rather than the absolute carbon content.*

Element	Theoretical wt% of $Cu_3(C_6O_6)$	Wt%
Cu	53.15	39.41
O	26.76	21.62
C	20.09	38.97
Total:	100.00	100.00

Table S3. Quantitative XPS atomic composition of the $Cu_3(C_6O_6)$ film compared with the theoretical atomic composition of $Cu_3(C_6O_6)$.

Element	Theoretical atomic% of $Cu_3(C_6O_6)$	Atomic%
C1s	40.0	40.52
O1s	40.0	41.16
Cu2p	20.0	18.32
Total:	100.00	100.00

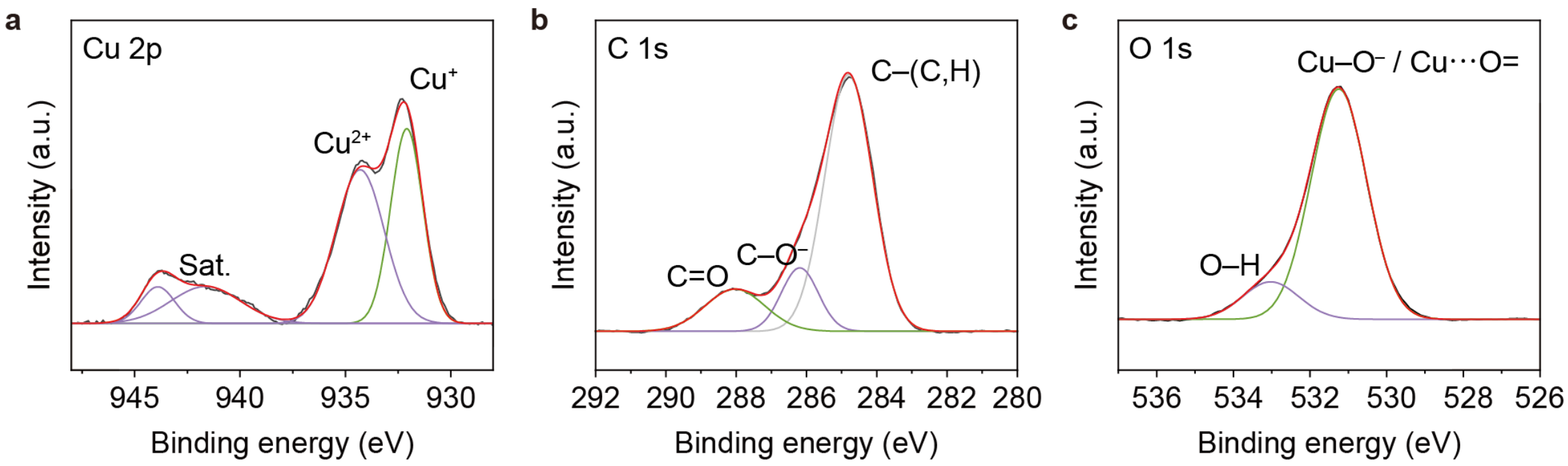

Fig. S12. High-resolution XPS spectra of $Cu_3(C_6O_6)$: (a) Cu 2p, (b) C 1s, and (c) O 1s. Deconvoluted peak components are shown together with the fitted curves.[5]

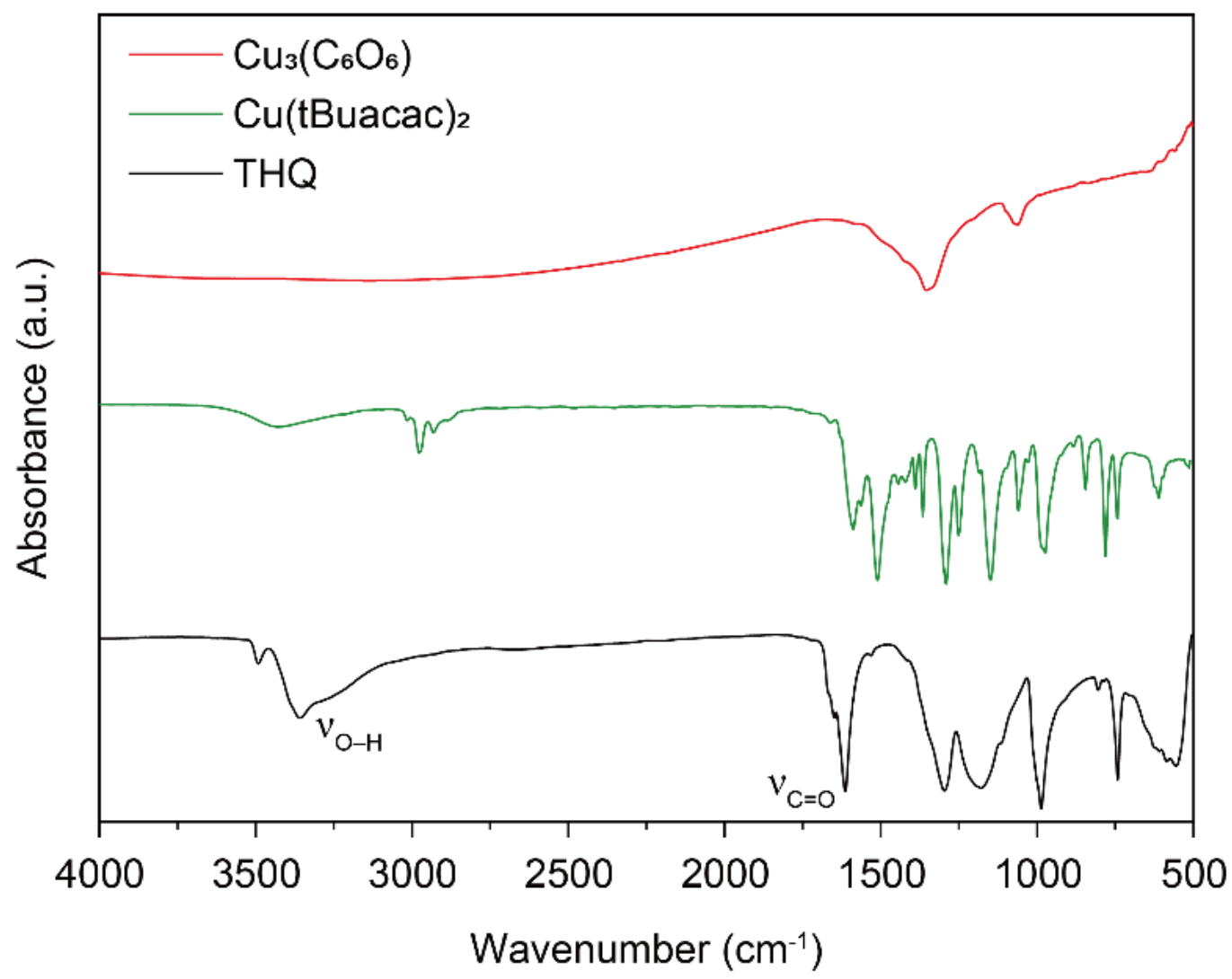

Fig. S13. ATR FT-IR spectra of the $Cu_3(C_6O_6)$ film measured on a 50 nm Au/Si substrate, along with $Cu(tBuacac)_2$ and THQ.

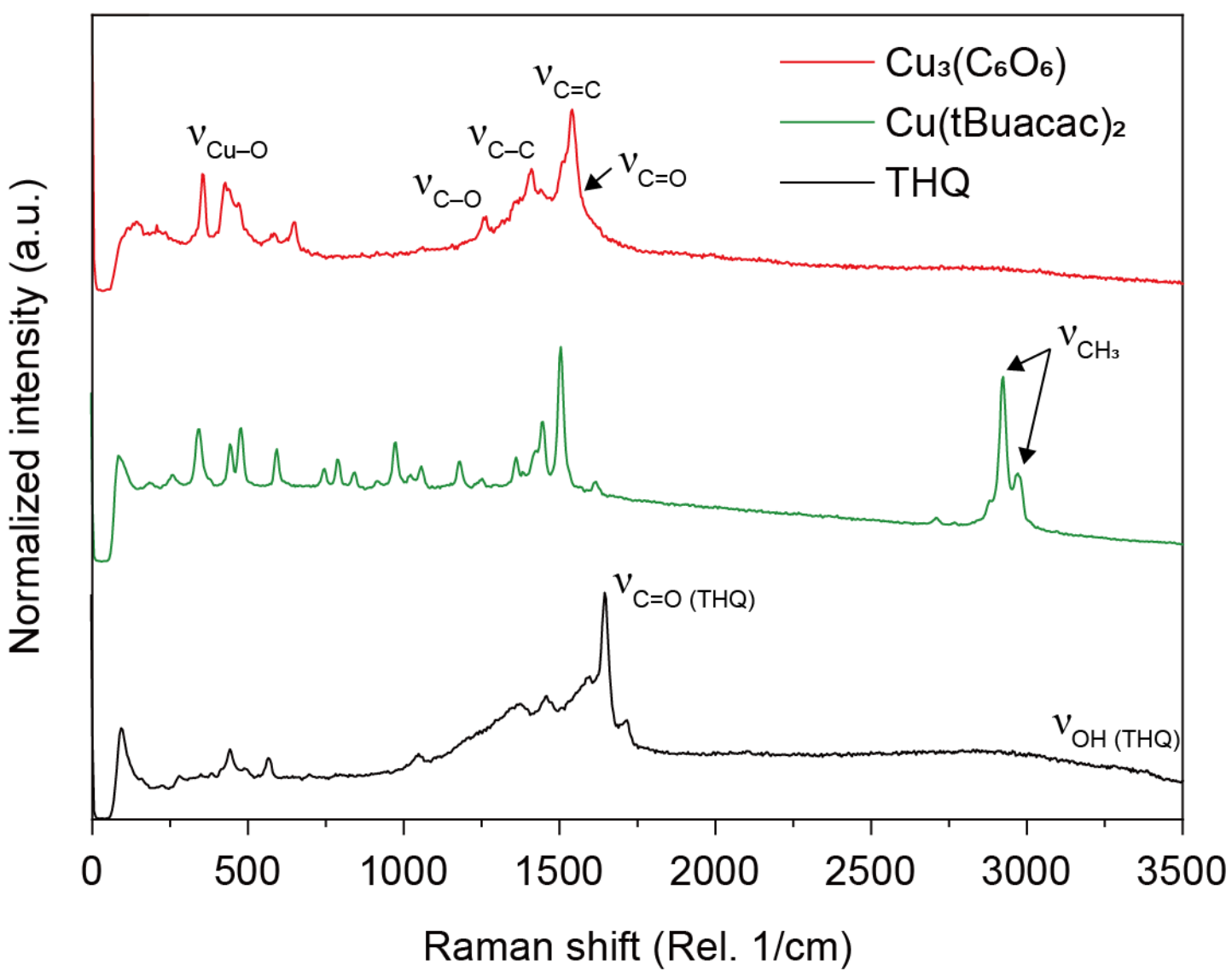

Fig. S14. Raman spectra of the $Cu_3(C_6O_6)$ film, $Cu(tBuacac)_2$, and THQ. Representative vibrational modes assigned to the $Cu_3(C_6O_6)$ film are labeled.

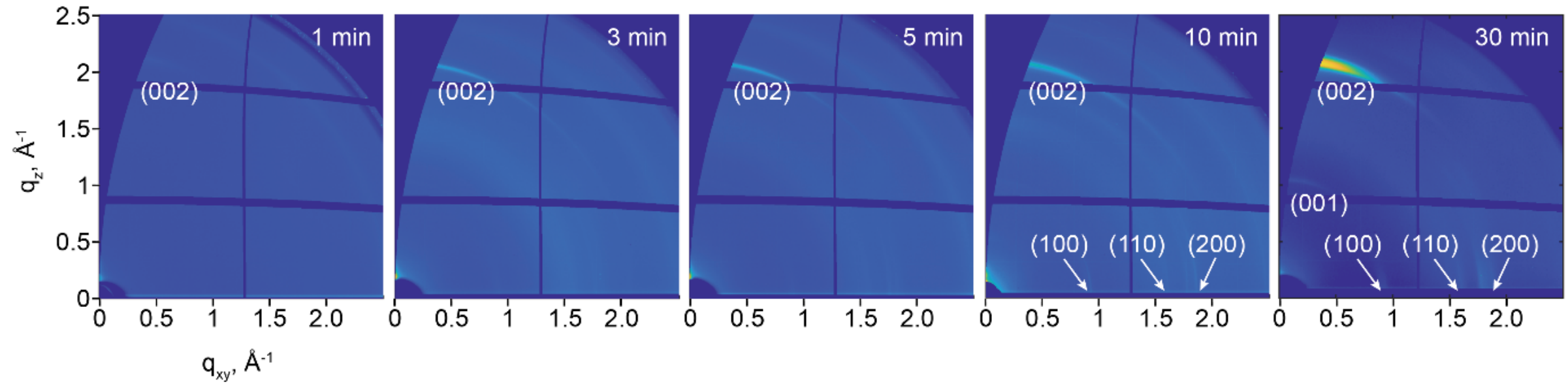

Fig. S15. Time-dependent ex situ GIWAXS patterns of $Cu_3(C_6O_6)$ films synthesized using $Cu(tBuacac)_2$ as the metal precursor; reaction times: 1, 3, 5, 10, and 30 min.

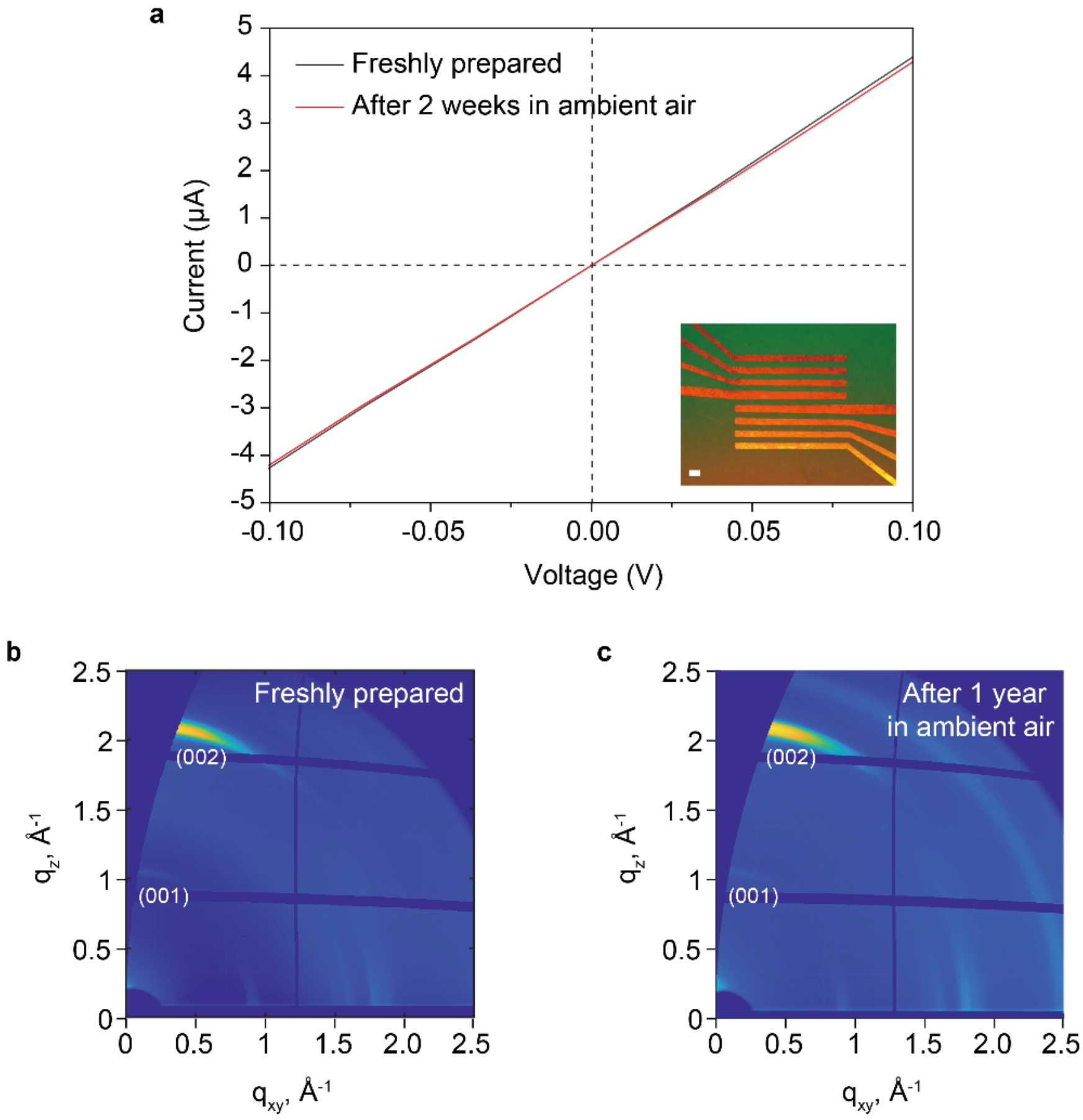

Fig. S16. Stability of the $Cu_3(C_6O_6)$ film under ambient conditions. (a) I-V characteristics of the freshly prepared film and the film stored under ambient conditions for 14 days. GIWAXS patterns of (b) the freshly prepared film and (c) the film stored under ambient conditions for 1 year. (Scale bar: 50 μm)

Table S4. Summary of reported single-step CVD syntheses of coordination polymer thin films, including CP name, metal precursor, ligand precursor, substrate, heating temperature, additive, and reference.

Name of CP	Metal precursor	Ligand precursor	Substrate	Heating temperature	Additive	Ref
-	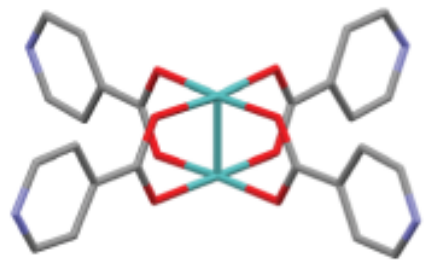 $Mo_2(INA)_4$ (INA: isonicotinate)		SiO_2/Si Glass	325 °C	-	6
$Fe_n(bim)_{2n}$	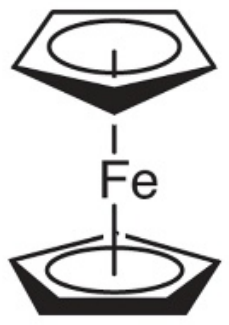 Ferrocene	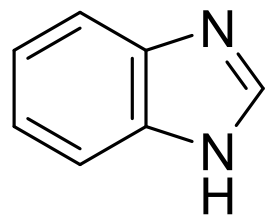 Benzimidazole	SiO_2/Si Sapphire	400 °C (metal) 150 °C (ligand)	-	7
$Fe_3(C_6O_6)$ $Fe_3(C_6S_6)$ $Cu_3(C_6O_6)_2$	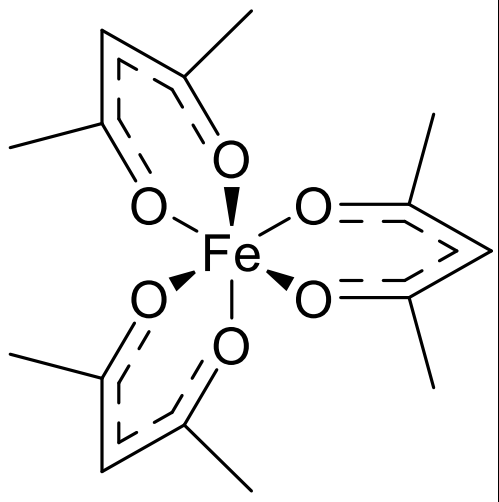 $Fe(acac)_3$ (acac: acetylacetonate) 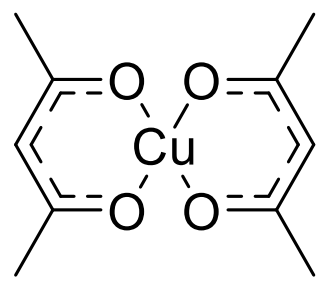 $Cu(acac)_2$	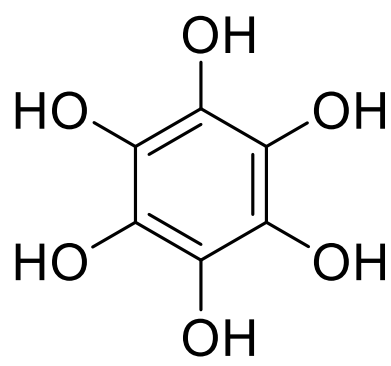 Hexahydroxybenzene 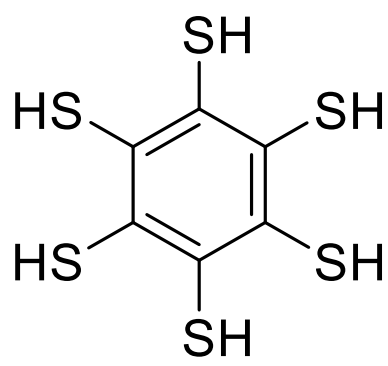 Benzenehexathiol	SiO_2/Si	105 °C (metal) 200 °C (ligand)	$NH_3 \cdot H_2O$	8
$M_3(C_6S_6)$ *(M = Cu, Ni, Co, Pd, V, Pt)*	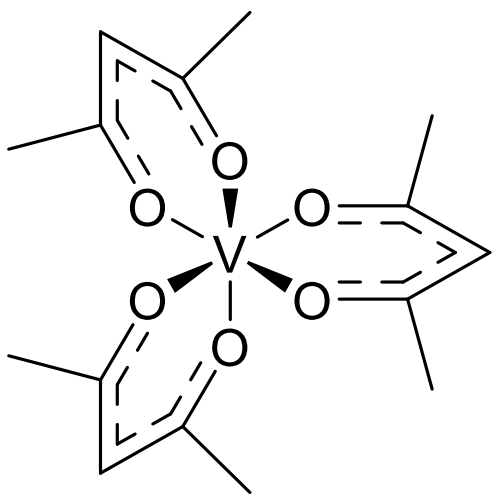 $V(acac)_3$ 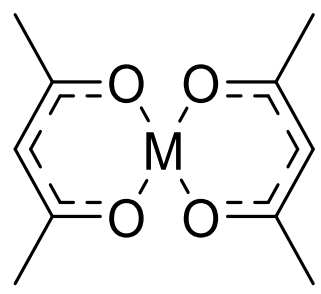 $M(acac)_2$ *(M = Cu, Ni, Co, Pd, Pt)*	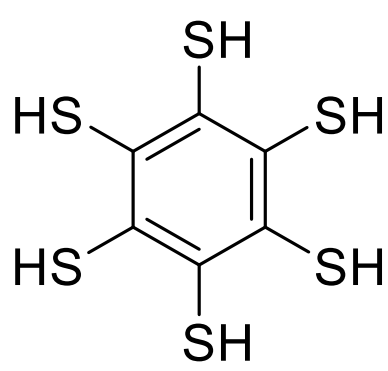 Benzenehexathiol	Ga droplet on W foil	120–165 °C 1000 °C (substrate)	-	9

$Cu_3(C_6S_6)$	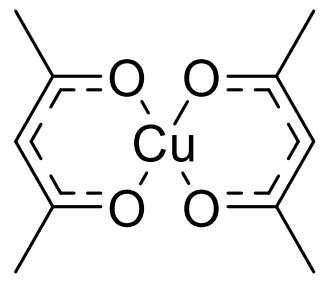 Cu(acac)$_2$	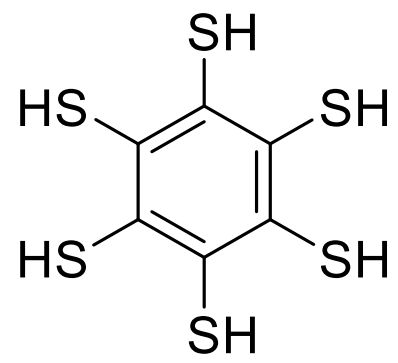 Benzenehexathiol	Glass	120 °C (metal) 80 °C (ligand)	-	10
$Cu_3(C_6O_6)_2$	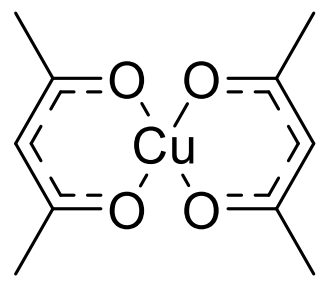 Cu(acac)$_2$	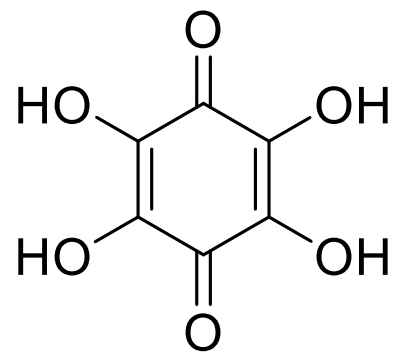 Tetrahydroxy-1,4-benzoquinone	SiO_2/Si	180 °C	-	1
INT	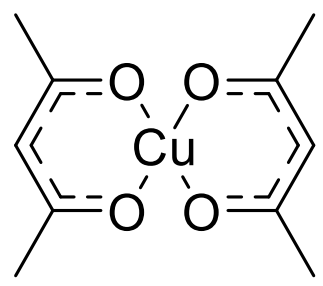 Cu(acac)$_2$	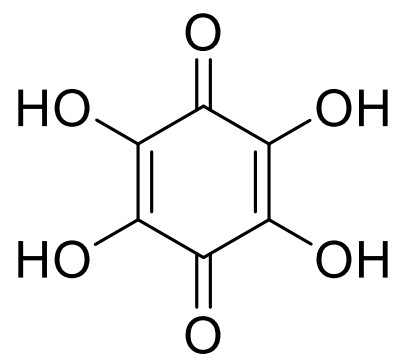 Tetrahydroxy-1,4-benzoquinone	SiO_2/Si	140 °C	-	11
**$Cu_3(C_6O_6)$** **This work**	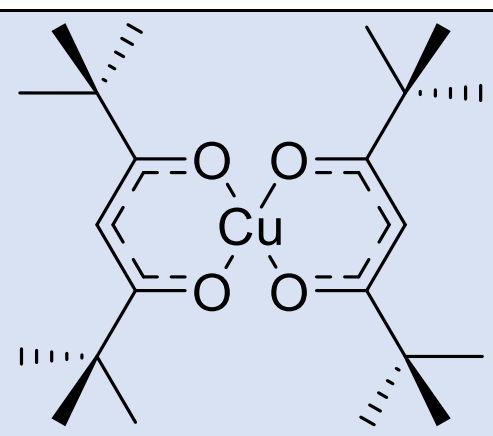 Cu(tBuacac)$_2$ (tBuacac: tert-butylacetoacetate)	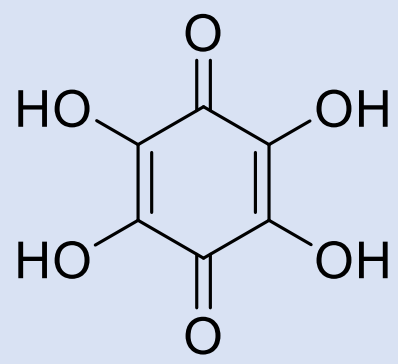 Tetrahydroxy-1,4-benzoquinone		140 °C	-	

References

(1) M. Choe, J. Y. Koo, I. Park, H. Ohtsu, J. H. Shim, H. C. Choi and S. S. Park, *J. Am. Chem. Soc.*, 2022, **144**, 16726–16731.

(2) Z. Wang, P. St. Petkov, J. Zhang, B. Liang, S. Revuelta, K. Xiao, K. Tiwari, Q. Guo, Z. Li, J. Zhang, H. Qi, S. Zhou, U. Kaiser, T. Heine, E. Cánovas, S. S. P. Parkin, X. Feng and R. Dong, *Adv. Funct. Mater.*, 2024, **34**, 2404680.

(3) Z. Gao, H. Ma, S. Yuan, H. Ren, Z. Ge, H. Zhu, W. Guo, F. Ding and W. Zhao, *Appl. Surf. Sci.*, 2022, **601**, 154187.

(4) M. Hua, B. Xia, M. Wang, E. Li, J. Liu, T. Wu, Y. Wang, R. Li, H. Ding, J. Hu, Y. Wang, J. Zhu, H. Xu, W. Zhao and N. Lin, *J. Phys. Chem. Lett.*, 2021, **12**, 3733–3739.

(5) R. Zhang, J. Liu, Y. Gao, M. Hua, B. Xia, P. Knecht, A. C. Papageorgiou, J. Reichert, J. V. Barth, H. Xu, L. Huang and N. Lin, *Angew. Chem. Int. Ed.*, 2020, **59**, 2669–2673.

(6) F. J. Claire, M. A. Solomos, J. Kim, G. Wang, M. A. Siegler, M. F. Crommie and T. J. Kempa, *Nat. Commun.*, 2020, **11**, 5524.

(7) L. Luo, L. Hou, X. Cui, P. Zhan, P. He, C. Dai, R. Li, J. Dong, Y. Zou, G. Liu, Y. Liu and J. Zheng, *Nat. Commun.*, 2024, **15**, 3618.

(8) J. Liu, S. Fu, Y. Fu, Y. Chen, K. Tadayon, M. Hambsch, D. Pohl, Y. Yang, A. Müller, F. Zhao, S. C. B. Mannsfeld, L. Gao, M. Bonn, X. Feng and R. Dong, *J. Am. Chem. Soc.*, 2025, **147**, 18190–18196.

(9) J. Liu, Y. Chen, X. Huang, Y. Ren, M. Hambsch, D. Bodesheim, D. Pohl, X. Li, M. Deconinck, B. Zhang, M. Löffler, Z. Liao, F. Zhao, A. Dianat, G. Cuniberti, Y. Vaynzof, J. Gao, J. Hao, S. C. B. Mannsfeld, X. Feng and R. Dong, *Nat. Synth.*, 2024, **3**, 715–726.

(10) J. Ogle, N. Lahiri, C. Jaye, C. J. Tassone, D. A. Fischer, J. Louie and L. Whittaker-Brooks, *Adv. Funct. Mater.*, 2021, **31**, 2006920.

(11) M. Choe, S. S. Park and H. C. Choi, *Inorg. Chem.*, 2024, **63**, 22662–22666.
